# Digital access, digital health information engagement, and self-reported preventive behavior among rural adults in Guizhou, China: media-use ecologies and cross-sectional associations

**DOI:** 10.3389/fpubh.2026.1794204

**Published:** 2026-06-24

**Authors:** Yuxiao Lyu, Chaozhong Luo

**Affiliations:** School of Digital Media, Guiyang Institute of Information Science and Technology, Guiyang, Guizhou, China

**Keywords:** digital access, digital health communication, digital health engagement, media-use ecologies, rural China, self-reported preventive behavior

## Abstract

**Background:**

Digital health education may help reduce health-information inequality in underdeveloped rural areas, but evidence remains limited on how rural residents encounter health information across different media environments and how digital access, usability, engagement, and self-reported preventive behavior are interrelated. This study examined media-use ecologies and cross-sectional associations among digital access and skills, digital health information engagement, and self-reported preventive behavior among rural adults in Guizhou, China.

**Methods:**

A cross-sectional survey was conducted among 1,265 adult rural residents recruited from five selected counties/districts in Guizhou Province using a multistage non-probability sampling design. Latent class analysis was used to characterize health-information media-use ecologies based on nine indicators of information channels and social media platforms. Regression-based cross-sectional association models examined associations among digital access and skills, perceived ease of understanding digital health content, lower operational difficulty, digital health information engagement, attitudes and willingness toward health education, and self-reported preventive behavior, adjusting for sex, age, education, income, and media-use ecology.

**Results:**

Five media-use ecologies were identified, reflecting different combinations of offline interpersonal/professional channels, traditional media, and digital platforms. Residents in omnichannel and short-video/social-platform-centered ecologies reported higher digital health information engagement, whereas those in the offline village doctor/traditional channels ecology reported the lowest engagement. Higher digital access and skills were associated with stronger engagement, and this association was attenuated after accounting for perceived ease of understanding and lower operational difficulty. Greater engagement was associated with more frequent self-reported preventive behavior, and this association was attenuated after accounting for attitudes toward health education and willingness to adopt new forms of health education.

**Conclusion:**

In this non-probability adult sample from selected rural sites in Guizhou, digital health inequality was reflected not only in unequal access to devices and networks, but also in differences in understanding, usability, engagement, and self-reported preventive behavior. The findings should be interpreted as cross-sectional associations among field-feasible indicators rather than evidence of causal mechanisms.

## 1 Introduction

### 1.1 Global context: digital health, digital divides and health inequalities

Over the past decade, the rapid diffusion of mobile internet, smartphones and cloud computing has turned “digital health” into an integral component of national health systems worldwide (1, 2). In its Global strategy on digital health 2020–2025, the World Health Organization (WHO) calls for the use of mobile applications, mini-programs, telemedicine platforms, wearables and social media, among other digital tools, to enhance the accessibility and quality of health information and services, advance universal health coverage, and uphold the equity principle of “leaving no one behind” (1).

Digital media are now embedded in everyday health-information seeking and self-management, and, when appropriately supported, can complement traditional health education and improve access in remote areas (3–7).

However, digital technologies are not inherently equitable. “Digital inclusion” is increasingly recognized as a new social determinant of health: whether people have reliable access to devices and networks, possess the skills and digital literacy needed to use them, and are able to derive tangible benefits from digital applications largely shapes who can share the dividends of digital health (8–10). Digital health equity research commonly distinguishes inequalities in access, skills, and returns from digital health use, and these layers may accumulate over time (11–14).

Existing evidence points to the dual potential of digital health. On the one hand, it is expected to help narrow urban–rural and regional health gaps; on the other hand, it may exacerbate inequalities in settings where infrastructure is weak and digital skills are limited—particularly among older adults (15–17) and socioeconomically disadvantaged groups with lower education and income (18, 19). Understanding how digital health reshapes health behaviors in specific contexts, and whether the resulting layered digital divides translate into new forms of health inequality, has therefore become a pressing concern for international research and public health practice on digital health (3, 5, 15–19), and provides the overarching backdrop for the present study.

### 1.2 Theoretical and conceptual framework: KAP model, media use and digital health literacy

To explain how these layered digital inequalities may translate into health-related behavior, the present study draws on the knowledge–attitude–practice (KAP) framework together with research on media use and eHealth literacy. In research on health behavior and health education, the KAP model is one of the most widely used theoretical frameworks (20–22). The model typically assumes that individuals first build a knowledge base about health topics through exposure to information and learning; on this basis, they develop attitudinal tendencies such as value judgements, risk perceptions and self-efficacy, which, when motivation and resources allow, are eventually translated into concrete preventive or treatment behaviors (20, 21, 23–26).

Within this framework, media exposure remains an important condition for knowledge formation, attitude development, and behavior. In digital environments, however, the issue is no longer exposure alone; it also matters through which media ecologies people encounter health information and whether those environments support meaningful engagement (27–32).

Alongside media use, digital health literacy shapes whether individuals can search for, understand, appraise, and use online health information. In rural settings, this competency is closely tied to the second-level digital divide (33–36).

Even where basic access exists, differences in the ability to understand and use digital health information may translate into unequal behavioral and health benefits (34, 35, 37–43), corresponding to second- and third-level digital divides.

Overall, the KAP framework remains useful for understanding health behavior, while digital media use and eHealth literacy have become increasingly important for explaining how people encounter, understand, and act on health information (20–25, 30, 38, 40, 41). However, most existing studies still rely on urban or special-population samples, use relatively simple indicators of media use or literacy, and rarely examine how digital access and skills, media-use ecology, comprehension barriers, and engagement jointly shape preventive behavior in disadvantaged rural settings (6, 7, 14, 30, 38, 43). These limitations are especially important in underdeveloped rural environments, where county- and village-level quantitative evidence remains scarce (14, 43).

### 1.3 China's rural and Guizhou context: health behavior challenges in a disadvantaged digital environment

In China, digital-health and digital-rural initiatives have expanded the use of digital technologies in primary care and health education. However, implementation remains uneven across urban and rural areas, and many rural communities still face shortages in health-service capacity and basic public-health delivery (15, 16, 44–47).

Many rural areas also face population aging and out-migration, meaning that a substantial share of residents may have limited formal education, weaker prior media experience, and greater difficulty navigating rapidly changing digital-health environments (45, 48–50).

Guizhou represents a disadvantaged digital environment in which mountainous geography, uneven development, constrained health resources, and regional disparities in digital conditions may jointly shape how rural residents access and use digital health information (51–57).

In this setting, offline trusted channels coexist with WeChat and short-video platforms, yet digital engagement and the ability to understand and use health information remain uneven. Quantitative evidence on how these conditions are associated with preventive behavior in underdeveloped western rural settings is still limited (47, 58–66).

### 1.4 Research gaps and contributions of this study

Synthesizing the above global and China-specific literature, at least three interrelated gaps remain insufficiently addressed.

First, although digital divide research has increasingly emphasized that inequality extends beyond physical access to devices and networks, fewer studies have examined how digital access and skills are associated with meaningful engagement with digital health information in disadvantaged rural settings (8, 11–14). Existing empirical work often focuses either on access conditions or on general digital literacy, but pays less attention to how these capacities are associated with actual health-information use and participation in disadvantaged rural settings (37, 38, 43, 67).

Second, while the KAP framework and related models have long been used to explain how cognition, attitudes, and practices are connected in health education (20–25), quantitative studies in rural digital health contexts have seldom incorporated comprehension barriers and operational difficulty as functional factors that may be associated with the relationship between digital access/skills and engagement (30, 38, 67). Yet in low-resource and older rural populations, the ability to understand and operate digital health information may be a crucial condition for turning nominal access into actual benefit (33, 35, 36, 48–50, 68).

Third, although prior studies have separately discussed digital inequality, digital literacy, media use, and health behavior, fewer have examined these elements within an integrated empirical framework in underdeveloped rural environments (8, 11–14, 30, 38). In particular, limited quantitative evidence is available on how digital access and skills, comprehension and operational ease, digital health information engagement, attitudes toward health education, willingness to adopt new forms of health education, and self-reported preventive behavior are interrelated among rural adults in western China (47, 58, 59, 66).

To address these gaps, the present study focuses on rural adults in Guizhou, a less developed province in western China characterized by uneven digital conditions and constrained grassroots health-service environments (51–57). The study examines cross-sectional associations among digital access and skills, comprehension and operational ease, digital health information engagement, attitudes toward health education, willingness to adopt new forms of health education, and self-reported preventive behavior. Media-use latent class is used to summarize residents' broader health-information environments and to contextualize the regression-based association analyses. In this way, the study offers a field-based account of digital health inequality in disadvantaged rural settings, focusing on how access, usability, engagement, attitudes/willingness, and self-reported preventive behavior are interrelated.

### 1.5 Study aims

Building on the above theoretical and contextual foundations, this study examines digital health information engagement and self-reported preventive behavior among rural adults in selected rural sites in Guizhou, China. The analysis focuses on cross-sectional associations among digital access and skills, perceived ease of understanding digital health content, lower operational difficulty, digital health information engagement, attitudes and willingness toward health education, and the self-reported frequency of preventive behavior.

The study has three analytical aims. First, it identifies health-information media-use ecologies among rural adults and describes how digital health information engagement differs across these ecologies. Second, it examines whether digital access and skills, perceived ease of understanding, and lower operational difficulty are cross-sectionally associated with digital health information engagement after accounting for sociodemographic characteristics and media-use ecology. Third, it examines whether digital health information engagement is cross-sectionally associated with the self-reported frequency of preventive behavior, and whether this association is attenuated after accounting for attitudes toward health education and willingness to adopt new forms of health education.

### 1.6 Research questions

Accordingly, the study addresses the following research questions:

RQ1: Media-use ecologies and digital health engagement.

What health-information media-use ecologies can be identified among rural adults in the selected rural sites, and how does digital health information engagement differ across these ecologies?

RQ2: Digital access, comprehension, operational ease, and engagement.

Are digital access and skills, perceived ease of understanding digital health content, and lower operational difficulty cross-sectionally associated with digital health information engagement after accounting for sociodemographic characteristics and media-use ecology?

RQ3: Engagement, attitudes/willingness, and self-reported preventive behavior.

Is digital health information engagement cross-sectionally associated with the self-reported frequency of preventive behavior, and is this association attenuated after accounting for attitudes toward health education and willingness to adopt new forms of health education?

In this framework, latent class analysis is used to characterize health-information media-use ecologies. These classes are not treated as causal exposures; rather, they are used to contextualize the regression-based association analyses and to inform differentiated digital public health communication strategies.

## 2 Materials and methods

### 2.1 Study design and setting

This study used a cross-sectional questionnaire survey to examine digital health information access, digital health engagement and preventive behaviors among rural adults in Guizhou Province, Southwest China. Guided by the KAP model and informed by research on the digital divide and digital health literacy, we developed a hypothesized analytic framework specifying associations among digital access and skills, comprehension difficulties, digital health engagement, attitudes/intentions toward health education, and preventive behaviors, and used this framework to organize the questionnaire modules and subsequent association analyses.

Data were drawn from a community-based survey conducted between December 2024 and February 2025 in rural areas of several county-level jurisdictions in Guizhou Province.

### 2.2 Participants and data collection

#### 2.2.1 Sampling and fieldwork organization

This study used a multistage non-probability sampling design. The survey was conducted in five counties or districts of Guizhou Province, China: Dafang County, Jinsha County, Xishui County, Weining County, and Xixiu District of Anshun City. At the site-selection stage, these areas were chosen purposively to capture variation in local economic development, geographic context, and digital conditions in rural Guizhou.

Within each selected county or district, the research team further identified villages or communities that differed in local development conditions and field accessibility. In practice, one relatively better-off and one relatively less-developed village or community were selected in each site where feasible, so as to reflect contextual diversity in rural digital and health-service environments. This design was intended to capture variation across rural settings rather than to construct a strict probability-based provincial sampling frame.

At the participant-recruitment stage, adult residents were recruited mainly through on-site intercept convenience sampling in commonly used community settings, including village committee offices, township activity spaces, local markets, primary health facilities, and other public gathering places. Eligible residents were invited to complete the questionnaire either by scanning a QR code linked to the online survey or, when needed, with investigator assistance on site. To improve coverage beyond public-site recruitment, local community networks were also used to circulate the survey link among eligible residents who were temporarily unavailable at the survey site, and a limited number of household visits were conducted with local assistance to reach residents who were less likely to be captured at public collection points. For older adults, participants with limited smartphone skills, or those who did not complete the questionnaire independently, trained investigators provided interviewer-assisted completion after explaining the study and obtaining informed consent. This procedure was intended to reduce exclusion of residents with lower digital literacy during field implementation, although it could not fully eliminate the possibility of differential participation by level of digital access or device-use ability.

This sampling strategy was chosen for two reasons. First, the study aimed to examine association patterns and media-use differences across diverse rural contexts rather than to produce statistically representative province-wide prevalence estimates. Second, under real-world rural field conditions, complete resident lists were not available and strict probability sampling was not operationally feasible. To contextualize sample composition, we compared the analytic sample with available 2020 census descriptors aggregated across the five sampled counties/districts (Supplementary Table S8). These comparisons were used as descriptive benchmarks only and do not establish population representativeness, because the study used a multistage non-probability design and no probability-based weighting or post-stratification adjustment was applied. Accordingly, the resulting sample should be understood as a non-probability adult sample recruited from five selected rural counties/districts in Guizhou that is analytically informative for identifying media-use patterns and cross-sectional association patterns, but not statistically representative of the underlying populations in those areas or of rural Guizhou as a whole.

#### 2.2.2 Inclusion criteria and sample size

The target population of this study was rural adult residents living in the selected areas of Guizhou Province. For the present analysis, eligible participants were required to meet the following criteria: (1) usual residence in a village or community within the selected survey sites; (2) age 18 years or older; and (3) ability to understand the questionnaire and provide informed consent, either independently or with investigator assistance.

In total, approximately 1,400 questionnaires were distributed, and 1,343 valid responses were obtained. Of these, 1,265 questionnaires were completed by respondents aged 18 years or older and were included in the adult analytic sample used in this paper. A small number of questionnaires completed by respondents younger than 18 years were retained only for internal project purposes and were not included in the present analysis.

No formal *a priori* power calculation was performed. Instead, the target sample size was determined pragmatically based on the planned latent class analysis, regression-based cross-sectional association analyses, and the feasible scope of fieldwork across multiple rural sites. Before data collection, we aimed to recruit at least 1,000 adult respondents in total, with approximately 200 respondents targeted from each selected county or district. This target was intended to provide an analytically adequate sample for identifying media-use ecologies and examining cross-sectional association patterns within the study setting, rather than to achieve province-wide statistical representativeness. The final adult sample of 1,265 respondents was therefore considered sufficient for the planned latent class analysis (LCA) and regression-based analyses in this multi-site rural sample.

#### 2.2.3 Missing data and analytical sample

For descriptive statistics, we reported percentages or means after excluding missing values on the variable concerned (available-case analysis). The proportions of missing data for the main outcome variables and key explanatory variables were low; detailed information on missingness is provided in the notes to Supplementary Table S1.

For the LCA of health-information media-use ecology, we fitted models using listwise deletion on the nine binary indicators of health information channels and social media platforms (see Section 2.3.1), restricting the LCA sample to adult respondents with valid responses on all nine indicators.

For the regression-based cross-sectional association models, analyses were conducted using the available adult analytic sample with valid data on the variables included in each model. The main variables used in these models had low levels of missingness, as shown in Supplementary Table S1. For the ordered-outcome sensitivity models, missing data were handled according to the default procedures of the corresponding model specifications implemented in the software.

### 2.3 Measures and variables

The questionnaire was developed by the research team based on the study objectives and relevant domestic and international literature, and was finalized after several rounds of internal discussion and revision (20, 33). It covered modules on sociodemographic characteristics, media use, digital access and skills, difficulties in understanding and operating digital health information, digital health information engagement behaviors, attitudes toward and intentions to adopt health education, and preventive behaviors.

Because the survey was implemented in multi-site rural field settings with constraints on questionnaire length, respondent burden, and heterogeneous literacy and digital-use ability, several constructs in this study were operationalized using concise field-feasible indicators rather than full validated multi-item scales. These measures were intended to capture core functional dimensions relevant to digital health information access, comprehension, engagement, attitudes, willingness, and self-reported preventive behavior while remaining feasible for community-based data collection. Accordingly, the indicators used here should be interpreted as pragmatic proxy measures for examining cross-sectional associations in a disadvantaged rural setting, rather than as exhaustive measurement instruments for the full underlying constructs. Item wording, coding direction, and the detailed mapping between questionnaire item codes/analytic variable names and the readable construct labels used in the manuscript are provided in Supplementary Data Sheet 1 and Supplementary Table S9.

This study draws on the staged logic of the KAP framework but adapts it to a digital health education context. In this framework, digital access and skills and comprehension/operational ease are treated as preconditions for digital health information use; engagement, attitudes, and willingness are treated as intermediate correlates of health-information use; and self-reported preventive behavior is treated as a proximal self-reported practice-related outcome. This framework was used to guide the selection, ordering, and interpretation of variables in the association analyses.

#### 2.3.1 Media-use indicators for latent class analysis

To characterize patterns of media use for obtaining health information, we constructed the observed indicators for LCA from two sets of variables:

1. Health information channels (Q7; five binary indicators): whether respondents reported obtaining health information through television, radio, mobile phone/internet, village doctors or village clinics, and relatives or friends.
2. Social media platforms (Q14; four binary indicators): whether respondents reported using WeChat, Douyin, Kuaishou or Weibo to obtain health information.

Each item was recoded into a binary variable, with “0 = not used/rarely used” and “1 = used/frequently used.” Together, these nine indicators capture the combined use of traditional mass media, offline interpersonal/professional channels and digital platforms in the dissemination of health information, and serve as the basis for identifying media-use latent classes.

#### 2.3.2 Digital health information engagement

Digital health information engagement (engagement_index) was operationalized as the sum of three binary indicators capturing whether respondents had ever used a mobile phone or computer to obtain health information (Q12), used mobile health applications (Q13), and participated in online health education content such as courses or videos (Q15). The resulting index ranged from 0 to 3, with higher scores indicating broader engagement with digital health information. This index was intended as a concise breadth-of-engagement measure for rural field settings rather than a comprehensive scale of engagement intensity or quality. Overall descriptive statistics for this index are presented in Supplementary Table S1, and differences across media-use latent classes are shown in Table 1 and Supplementary Table S2.

**Table 1:** Digital health information engagement across media-use classes.

Media-use latent class (*K* = 5)	*n*	Engagement index (mean ±SD)	Median (IQR)	Q12 used (%)	Q13 used (%)	Q15 used (%)
Mobile–WeChat/Douyin light-use	513	1.18 ± 1.18	1 (0–2)	48.5	40.4	28.8
Offline village doctor/traditional channels (low-digital)	182	0.47 ± 0.87	0 (0–1)	18.1	19.8	9.3
Omnichannel high-engagement	125	2.25 ± 1.01	3 (2–3)	85.6	69.6	69.6
Short video–social platforms + TV	287	1.99 ± 1.06	2 (1–3)	75.3	70.7	53.0
Village doctor/relatives + short video social	158	1.07 ± 1.10	1 (0–2)	43.0	38.6	25.3

The engagement_index ranges from 0 to 3 and is constructed from three binary items: Q12, Q13, and Q15. Percentages are within-class percentages based on modal class assignment. Median (IQR) was calculated from the individual-level dataset with modal class labels. Overall differences across classes were assessed using the Kruskal–Wallis test for the engagement_index and Pearson chi-square tests for Q12, Q13, and Q15.

#### 2.3.3 Digital access and skills index (AccessSkills)

The digital access and skills dimension was summarized using three items (Q8–Q10) covering device familiarity, internet use, and local network coverage. Each item was first coded from better to worse conditions and then reverse-coded so that higher values represented better digital access conditions and stronger usage skills. The three reverse-coded items were standardized separately (*z*-scores) and then averaged to construct a continuous index, AccessSkills. Higher values of AccessSkills indicate that respondents are more proficient in operating devices, use mobile phones/computers to access the internet more frequently, and live in villages with relatively better network coverage. This composite index should be understood as a pragmatic summary of basic digital access conditions and self-reported operational familiarity in the study setting, rather than as a full standardized measure of digital literacy or digital competence.

#### 2.3.4 Ease of understanding and operational difficulty

To capture comprehension-related and operational barriers in digital health information use, we used two four-point items: ease of understanding digital health content (Q16_rev) and difficulty using a phone or computer to obtain health information (Q17_rev, reverse-coded so that higher scores indicate fewer difficulties). Both items were reverse-coded so that higher scores indicated fewer barriers. These variables were treated as concise proxy indicators rather than full multidimensional measures of digital health literacy. Item wording and coding are provided in Supplementary Data Sheet 1.

#### 2.3.5 Attitudes toward health education and willingness to adopt new forms of access

Two four-point items were used to represent attitudes toward health education and willingness to adopt more convenient ways of obtaining health information: perceived helpfulness of health education (Q26_rev) and willingness to try more convenient access modes (Q29_rev). Both were reverse-coded so that higher scores indicated more positive attitudes or stronger willingness. In the regression-based association analyses, these variables were used to examine whether the association between digital health information engagement and self-reported preventive behavior was attenuated after accounting for attitudes toward health education and willingness to adopt new forms of health education (22). Item wording and coding are provided in Supplementary Data Sheet 1.

#### 2.3.6 Self-reported preventive behavior outcome

The outcome was measured using Q23, a single four-category item asking whether respondents take proactive measures to prevent disease in daily life. Responses were reverse-coded so that higher values indicated more frequent self-reported preventive behavior. Given the constraints of multi-site rural fieldwork and respondent burden, this item was used as a concise, field-feasible indicator of broad preventive behavioral tendency/frequency rather than as a comprehensive validated multi-item scale. It does not distinguish among specific types of preventive practices (for example, diet-related actions, hygiene routines, physical activity, or routine health check-ups) and should therefore be interpreted as a pragmatic proxy outcome. The exact item wording and coding are provided in Supplementary Data Sheet 1.

#### 2.3.7 Covariates (sex, age, education, and monthly income)

Sociodemographic variables included sex, age, education and monthly income, which were used as covariates in the main regression-based association models. Sex was coded as “male” or “female.” Age was categorized into six groups: 18–25, 26–30, 31–40, 41–50, 51–60 and >60 years. Education was classified into seven levels: no formal schooling, primary school, junior high school, senior high school, technical secondary school, college (2–3 years) and bachelor's degree or above. Monthly income was grouped into four categories: < 2,000 CNY, 2,000–4,999 CNY, 5,000–9,999 CNY and ≥10,000 CNY. Descriptive distributions of these variables for the adult sample are summarized in Table 2.

**Table 2:** Participant characteristics (adult analytic sample, *N* = 1,265).

Variable	Category	*n*	%
Gender (*N* = 1,265)	Male	619	48.9
	Female	646	51.1
Age group (*N* = 1,265)	18–25	295	23.3
	26–30	113	8.9
	31–40	191	15.1
	41–50	240	19.0
	51–60	236	18.7
	>60	190	15.0
Education (*N* = 1,265)	No formal schooling	147	11.6
	Primary school	277	21.9
	Junior high school	306	24.2
	Senior high school	109	8.6
	Technical secondary school	58	4.6
	College (2–3 years)	140	11.1
	Bachelor's degree or above	228	18.0
Monthly income (*N* = 1,265)	< 2,000 CNY	506	40.0
	2,000–4,999 CNY	540	42.7
	5,000–9,999 CNY	194	15.3
	≥10,000 CNY	25	2.0
Residence (*n* = 1,225)	Dafang county	256	20.9
	Jinsha county	184	15.0
	Weining county	309	25.2
	Xishui county	191	15.6
	Anshun Xixiu rural area	280	22.9
	Other	5	0.4
Occupation (*n* = 1,225)	Farming (agriculture/forestry/animal husbandry etc.)	375	30.6
	Self-employed	261	21.3
	Student	209	17.1
	Unemployed/retired	222	18.1
	Others (e.g., village cadres, teachers, workers)	158	12.9
Ethnicity (*n* = 1,225)	Han	736	60.1
	Yi	150	12.2
	Miao	35	2.9
	Buyi	264	21.6
	Other ethnic groups	40	3.3

The adult analytic sample included 1,265 respondents; in total, 1,343 questionnaires were valid. Percentages for gender, age, education and income are based on this adult analytic sample (*N* = 1,265). Percentages for residence, occupation and ethnicity are based on valid responses for each variable (*n* as indicated), and therefore may not sum to 1,265 due to item non-response and rounding.

For exploratory interaction checks, we further constructed three binary grouping variables based on the above categories. Age was grouped as 18–40 vs. ≥41 years. Education was grouped as junior high school or below vs. senior high school or above. Monthly income was grouped as < 5,000 CNY vs. ≥5,000 CNY. These groupings were used only for exploratory interaction checks in regression models and were not treated as primary grouping variables for confirmatory subgroup analysis.

The cut-points for these groupings reflected a combination of substantive considerations and the empirical distribution of the sample. The age threshold around 40 years was close to the median age in this sample and roughly corresponded to an intergenerational turning point in family and work responsibilities in rural China. The distinction between junior high school or below and senior high school or above is consistent with the completion of compulsory education and common patterns of educational stratification in rural areas. The monthly income threshold of 5,000 CNY was chosen primarily with reference to the sample's income distribution and to create substantively meaningful groups for exploratory interaction checks. These groupings were intended to construct relatively parsimonious and interpretable strata under limited sample size, rather than to exhaustively capture all possible fine-grained socioeconomic differences.

### 2.4 Statistical analysis

All analyses were conducted using R version 4.5.1 (R Foundation for Statistical Computing, Vienna, Austria). Unless otherwise specified, statistical tests were two-sided with a significance level of α = 0.05, and 95% confidence intervals (CIs) were reported.

The primary analytical strategy consisted of three components. First, latent class analysis was used to characterize health-information media-use ecologies across nine indicators of information channels and social media platforms. Second, differences in digital health information engagement across media-use classes were described to contextualize how rural adults encountered and engaged with health information across different media environments. Third, regression-based cross-sectional association models were used as the primary analyses to examine associations among digital access and skills, perceived ease of understanding digital health content, lower operational difficulty, digital health information engagement, attitudes and willingness toward health education, and the self-reported frequency of preventive behavior, adjusting for sex, age, education, monthly income, and media-use ecology.

**Figure 1:**
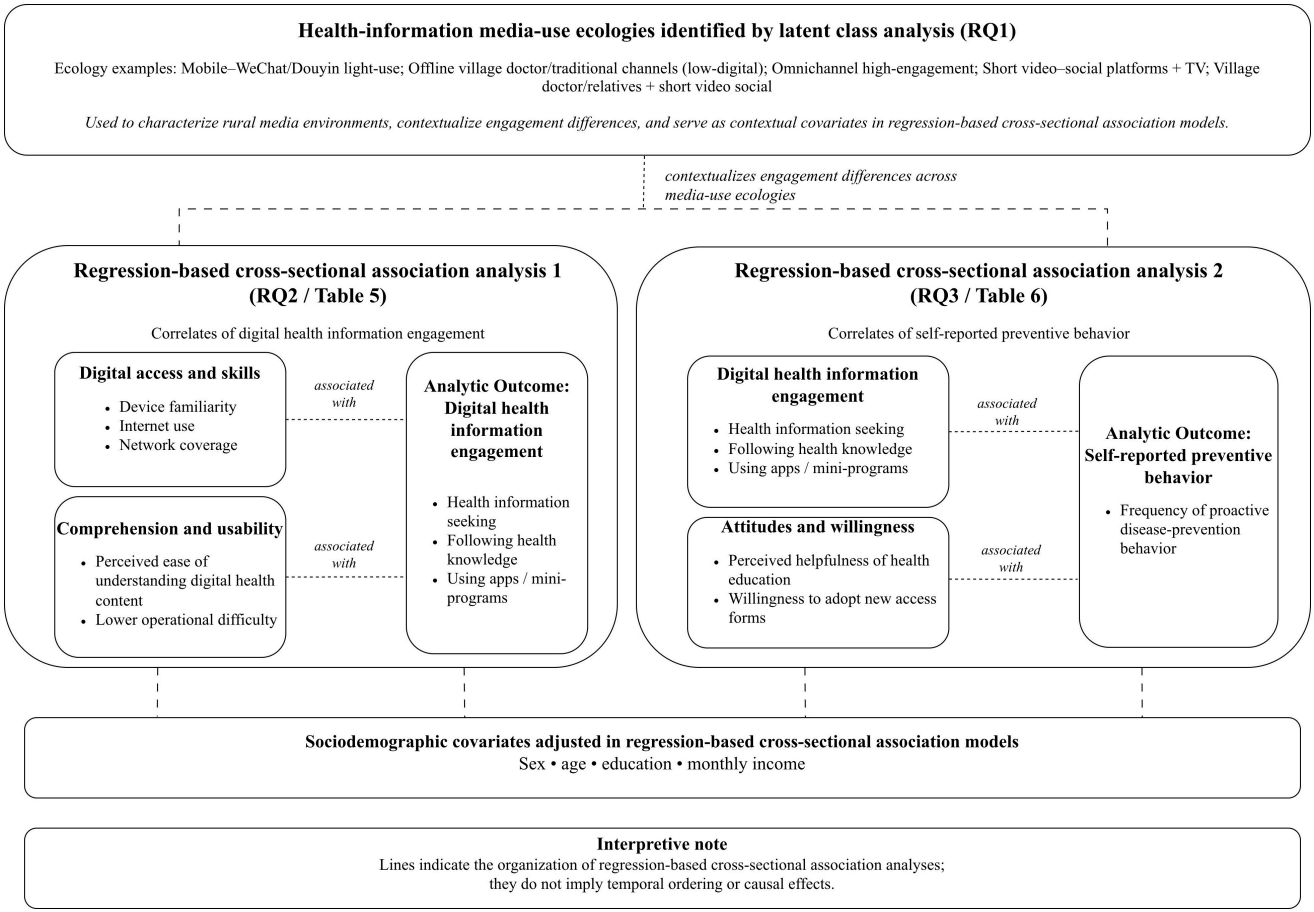
Conceptual framework for LCA-informed regression-based cross-sectional association analyses among media-use ecologies, digital access, digital health information engagement, and self-reported preventive behavior. The figure summarizes how the study variables were organized for LCA-informed regression-based cross-sectional association analyses. Latent class analysis was used to characterize health-information media-use ecologies, which were used to contextualize engagement differences and to serve as contextual covariates in the regression-based models. Two regression-based cross-sectional association modules were then used to examine correlates of digital health information engagement and correlates of self-reported preventive behavior, respectively. Sociodemographic characteristics were adjusted as covariates. Lines indicate the organization of the association analyses and do not imply temporal ordering or causal effects.

The conceptual framework shown in Figure 1 was used to organize the study variables and regression-based cross-sectional association analyses. This analytical strategy was chosen to align the statistical approach with the cross-sectional design and field-feasible measurement structure of the study.

#### 2.4.1 Latent class analysis of health-information media-use ecology

Based on the nine binary media-use indicators described in Section 2.3.1, we fitted latent class models with *K* = 2–5 classes using a conditional independence specification for binary outcomes (LCA). Multiple sets of random starting values and iterations were used to reduce the risk of convergence to local rather than global (or near-global) optima.

To select the number of classes, we jointly considered log-likelihood values, information criteria (AIC/BIC), likelihood-based fit statistics (*G*^2^/χ^2^), entropy, the size of the smallest class, and substantive interpretability of the class profiles. The final number of classes for the main analysis was chosen by balancing statistical fit and parsimony. Each respondent's class membership was assigned by maximum posterior probability (modal assignment), and the posterior probabilities were retained for subsequent soft-class robustness analyses. LCA was implemented using the poLCA package in R (69).

For the selected latent class solution, we reported class proportions, item-response probabilities, entropy, average posterior probabilities by assigned class, the mean of the maximum posterior probability, and classification uncertainty calculated as 1 minus the maximum posterior probability. These diagnostics were used to evaluate the degree of separation among classes and the uncertainty associated with modal class assignment. The posterior probabilities were also retained for soft-class robustness analyses.

#### 2.4.2 Analysis of digital health engagement across media-use classes

We examined differences in digital health engagement across media-use classes using two complementary analytical approaches.

1. Using the engagement index (0–3) as an ordinal outcome, we applied the Kruskal–Wallis rank-sum test to assess overall differences across the five latent classes. When the overall test was statistically significant, we carried out pairwise Wilcoxon rank-sum tests with Holm adjustment for multiple comparisons to examine between-class differences in more detail.
2. For each of the three binary engagement items (Q12, Q13, Q15), we constructed contingency tables of “class × engagement (yes/no)” and used Pearson χ^2^ tests to examine whether the prevalence of each specific engagement behavior differed across the five classes.

The main analyses were based on hard class assignment (modal assignment). As a robustness check, Supplementary Table S5 presents soft-class analyses in which each respondent's contribution to each class is weighted by their posterior class membership probabilities. Under this approach, we recalculated the engagement index and the three engagement behaviors as class-wise weighted means, and compared classes using bootstrap CIs, thereby taking into account uncertainty in class assignment.

#### 2.4.3 Role of media-use latent class in the association analyses

Because health-information use is typically organized as a combination of channels rather than isolated platform behaviors, latent class analysis was used to summarize broader health-information media-use ecologies. The resulting media-use classes were not interpreted as causal exposures. Instead, they were used in two ways: first, as descriptive indicators of differentiated rural media environments, and second, as contextual covariates in the regression-based cross-sectional association models. This approach allowed the analysis to account for broader media-use ecology while keeping the main focus on digital access, comprehension and operational ease, digital health information engagement, and self-reported preventive behavior.

#### 2.4.4 Regression-based association models

Regression-based models were used as the primary analyses to examine the cross-sectional associations corresponding to RQ2 and RQ3. To examine associations with digital health information engagement, we fitted a sequence of multivariable models. First, engagement_index was regressed on AccessSkills, adjusting for sex, age, education, monthly income, and media-use class. Second, Q16_rev and Q17_rev were separately regressed on AccessSkills and the same covariates. Third, engagement_index was regressed on AccessSkills, Q16_rev, Q17_rev, and the covariates to examine whether the association between AccessSkills and engagement_index was attenuated after accounting for perceived ease of understanding and lower operational difficulty.

To examine associations with self-reported preventive behavior, Q23_rev was regressed on engagement_index and the covariates. Q26_rev and Q29_rev were then separately regressed on engagement_index and the same covariates. Finally, Q23_rev was regressed on engagement_index, Q26_rev, Q29_rev, and the covariates to examine whether the association between engagement_index and Q23_rev was attenuated after accounting for attitudes toward health education and willingness to adopt new forms of health education.

Linear regression models were used as the primary regression-based specification to facilitate comparison of coefficients across nested models and to estimate attenuation in associations. Because several outcomes were ordinal or index-based, ordered-outcome models were additionally used in sensitivity analyses where applicable. Results were interpreted as cross-sectional associations rather than as evidence of temporal ordering or causal mediation (70, 71).

All linear regression models were estimated with HC3 heteroskedasticity-consistent robust standard errors to reduce the sensitivity of statistical inference to potential heteroskedasticity in the cross-sectional survey data (72).

Because the regression-based analyses examined related parts of the conceptual framework in separate models, we additionally conducted a supplementary exploratory integrated observed-variable path model to describe whether the overall direction of associations was broadly coherent when the observed variables were considered within a single analytic framework. This model was used as a supplementary descriptive check rather than as a primary analysis, and the detailed model specification, fit indices, and standardized coefficients are reported in Supplementary Tables S6, S7.

#### 2.4.5 Exploratory interaction checks by age, education, and monthly income

As an exploratory extension of the regression-based association analyses, we examined whether two selected associations varied by age, education, and monthly income: the association between AccessSkills and engagement_index, and the association between engagement_index and Q23_rev. Three binary grouping variables were constructed: age group (18–40 vs. ≥41 years), education level (junior high school or below vs. senior high school or above), and monthly income (< 5,000 CNY vs. ≥5,000 CNY).

Interaction terms were added to regression models to test potential heterogeneity. Specifically, AccessSkills × group interactions were tested in models predicting engagement_index, and engagement_index × group interactions were tested in models predicting Q23_rev. These analyses were exploratory and were used to assess whether the main regression-based associations showed clear heterogeneity across major socioeconomic strata.

#### 2.4.6 Robustness analyses

We conducted several robustness analyses to assess whether the main findings were sensitive to ordered-outcome specification, construction of the AccessSkills index, and uncertainty in media-use class assignment.

1) Ordered-outcome specification

Because several key variables were ordinal or index-based, including engagement_index, Q16_rev, Q17_rev, Q23_rev, Q26_rev, and Q29_rev, ordered-outcome sensitivity models were used to examine whether the direction and statistical significance of the focal associations were consistent with the primary linear regression models.

2) Construction of the AccessSkills index

To examine whether the findings were sensitive to the construction of the digital access and skills measure, we replaced the composite AccessSkills index with its standardized component indicators, Q8_z, Q9_z, and Q10_z. These models assessed whether the associations involving perceived ease of understanding, lower operational difficulty, and digital health information engagement were similar when the three components of digital access and skills were entered separately rather than combined into a single index.

3) Uncertainty in media-use class assignment

Because latent class membership is probabilistic, we also examined whether the descriptive and association patterns were sensitive to the use of hard-class assignment. For the selected LCA solution, posterior class membership probabilities were retained, and soft-class robustness analyses were conducted using posterior-probability weighting. These results were compared with the modal hard-class assignment results to assess whether conclusions about media-use patterns and digital health information engagement were sensitive to classification uncertainty.

Summary results of the robustness analyses are presented in Supplementary Tables S4, S5, S10.

#### 2.4.7 Software

Data cleaning and statistical analyses were performed in R version 4.5.1 (R Foundation for Statistical Computing, Vienna, Austria). Latent class analysis was conducted using the poLCA package, and the exploratory integrated observed-variable path model was estimated using lavaan. Regression-based models were fitted using base R functions, and HC3 robust standard errors were computed using the sandwich and lmtest packages. Ordered-outcome sensitivity models and table preparation were conducted using standard R packages and office software.

#### 2.4.8 Use of AI-assisted tools

During manuscript preparation, ChatGPT (OpenAI, San Francisco, CA, USA) was used only in a limited and occasional auxiliary manner for minor English language editing and troubleshooting of R syntax or error messages. Its role was restricted to these supportive tasks and did not extend to data generation, variable selection, statistical model selection, analytic decision-making, interpretation of findings, or alteration of numerical results. All coding, statistical implementation, verification of outputs, and interpretation of results were performed and confirmed by the authors.

## 3 Results

Table 2 summarizes the characteristics of the adult analytic sample (*N* = 1,265). Women accounted for 51.1% of participants, and slightly more than half of the respondents were aged 41 years or older. Most participants had junior high school education or below, and monthly income was concentrated in the < 2,000 CNY and 2,000–4,999 CNY categories. Additional details on county/district of residence, occupation, and ethnicity are shown in Table 2.

To contextualize the obtained sample, the sample composition was compared with available 2020 census descriptors aggregated across the five sampled counties/districts. The full descriptive comparison is presented in Supplementary Table S8. These comparisons are used only as contextual benchmarks to aid interpretation and do not imply that the non-probability sample is statistically representative of the underlying populations in the sampled areas or of rural Guizhou as a whole.

### 3.1 Latent class analysis of media-use patterns

#### 3.1.1 Model selection and classification diagnostics

Based on the nine binary indicators (five health information channels from Q7 and four social media platforms from Q14), we fitted latent class models with *K* = 2–5 classes (Table 3). As the number of classes increased from 2 to 5, the model log-likelihood values became progressively larger, while the corresponding *G*^2^ and χ^2^ statistics decreased, and the AIC/BIC values showed an overall improving trend. In particular, the BIC decreased from approximately 12,319 for *K* = 2 to about 11,811 for *K* = 5, with the *K* = 5 model achieving the lowest BIC. The entropy values for the *K* = 3–5 models were all around 0.75 (0.80 for *K* = 3, 0.76 for *K* = 4 and 0.75 for *K* = 5), indicating acceptable class separation.

**Table 3:** Fit indices for latent class models with two to five classes.

Classes (*K*)	Log-likelihood	AIC	BIC	Gsq	*X* ^2^	Entropy	Smallest class proportion
2	−6,092.046	12,222.092	12,319.806	1,236.244	1,816.006	0.711	0.353 (35.3%)
3	−5,895.348	11,848.695	11,997.837	842.847	1,122.016	0.797	0.162 (16.2%)
4	−5,799.859	11,677.718	11,878.288	651.870	898.869	0.758	0.138 (13.8%)
5	−5,730.468	11,558.937	11,810.935	513.089	759.785	0.746	0.105 (10.5%)

Lower AIC and BIC indicate better relative fit. Entropy ranges from 0 to 1, with higher values indicating clearer class separation. The smallest class proportion is based on model-estimated class proportions. The five-class solution was retained based on BIC, interpretability, and class size.

The estimated proportion of the smallest class across the models was approximately 35.3%, 16.2%, 13.8%, and 10.5% for *K* = 2–5, respectively. Taking into account model fit indices, interpretability of the class profiles and class sizes, we retained the *K* = 5 solution as the preferred summary of health-information media-use ecologies in the sample.

For the selected five-class solution, classification diagnostics suggested acceptable but not perfect separation among classes. The average maximum posterior probability ranged from 0.779 in the Doctor/interpersonal + short-video class to 0.956 in the Omnichannel high-engagement class. Because latent class membership is probabilistic, posterior-probability weighted soft-class analyses were also conducted as robustness checks.

#### 3.1.2 Five media-use patterns: class labels and characteristics (*K* = 5)

For the selected five-class solution, Table 4 reports the conditional response probabilities and the estimated proportion of each class. On the basis of the probability combinations for different channels/platforms, with higher probabilities indicating a greater likelihood of using the corresponding channel/platform, we labeled and interpreted the five health-information media-use ecologies as follows.

**Table 4:** Conditional response probabilities and class proportions for the five-class LCA solution.

Class	Class proportion	TV	Radio	Internet/mobile	Village doctor	Family/friends	WeChat	Douyin	Kuaishou	Weibo
Class 1	0.374 (37.4%)	0.485	0.098	0.878	0.258	0.068	0.794	0.760	0.091	0.042
Class 2	0.143 (14.3%)	0.382	0.131	0.004	0.638	0.342	0.161	0.181	0.015	0.004
Class 3	0.105 (10.5%)	0.776	0.938	0.986	0.956	0.966	0.969	0.954	0.819	0.427
Class 4	0.241 (24.1%)	0.708	0.256	0.899	0.200	0.144	1.000	1.000	0.751	0.389
Class 5	0.137 (13.7%)	0.119	0.000	0.867	0.826	0.654	0.942	0.966	0.431	0.000

Values are Pr (indicator = 1|class), estimated from the selected five-class latent class model. Class proportions are model-estimated LCA proportions. Indicators include five health information sources (TV, radio, Internet/mobile, village doctor, and family/friends) and four social media platforms (WeChat, Douyin, Kuaishou, and Weibo). Probabilities are rounded to three decimals.

Class 1, “Mobile–WeChat/Douyin light-use” (≈37.4%), showed the highest probability of using mobile phone/internet (0.878), and relatively high probabilities of using WeChat and Douyin (0.794 and 0.760, respectively). By contrast, the probabilities of using village doctors/clinics, relatives/friends, and radio were relatively low. This class can be understood as a light digital media-use ecology in which mobile internet and mainstream social platforms are the main sources of health information, with comparatively limited reliance on offline traditional channels.

Class 2, “Offline village doctor/traditional channels (low-digital)” (≈14.3%), showed relatively higher probabilities of using village doctors/clinics (0.638), relatives/friends (0.342), and television (0.382), whereas the probability of using mobile phone/internet was close to zero (0.004), and the probabilities of using WeChat and Douyin were clearly low. This class represents a low-digital, offline-oriented ecology in which health information is more likely to be encountered through village doctors, interpersonal networks, and traditional media.

Class 3, “Omnichannel high-engagement” (≈10.5%), showed high probabilities for almost all channels, including television, radio, mobile phone/internet, village doctors, relatives/friends, WeChat, Douyin, Kuaishou, and Weibo. This class represents a multi-channel ecology with high exposure across traditional media, offline interpersonal/professional channels, and multiple digital platforms.

Class 4, “Short video–social platforms + TV” (≈24.1%), showed high probabilities of using mobile phone/internet (0.899), television (0.708), WeChat (1.000), Douyin (1.000), and Kuaishou (0.751), with a non-negligible probability of using Weibo (0.389). In contrast, the probabilities of using village doctors, relatives/friends, and radio were relatively low. This class is characterized by short-video and social platforms at the core, complemented by television as a traditional channel.

Class 5, “Village doctor/relatives + short video social” (≈13.7%), showed high probabilities of using village doctors (0.826), relatives/friends (0.654), mobile phone/internet (0.867), WeChat (0.942), and Douyin (0.966), with a medium probability of using Kuaishou (0.431). Compared with the low-digital offline class, this class reflects a hybrid ecology combining offline trusted intermediaries with digital social and short-video platforms.

These class labels provide contextual information for the subsequent regression-based association analyses and for interpreting differentiated digital health communication strategies. The class proportions reported in Table 4 are model-estimated LCA proportions; as expected, they differ slightly from the sample frequencies based on modal assignment used in the class-level descriptive analyses reported in Table 1.

### 3.2 Digital health information engagement across media-use classes

#### 3.2.1 Hard-assignment results based on modal class membership

Table 1 reports differences in digital health information engagement across the five media-use classes based on modal class assignment. Overall, the distribution of the engagement index (0–3) differed significantly across the five classes (Kruskal–Wallis χ^2^ = 261.48, df = 4, *p* < 0.001). Among them, the “Omnichannel high-engagement” class had the highest engagement index (2.25 ± 1.01): approximately 85.6% of individuals had ever actively searched for health information (Q12), 69.6% had proactively followed health knowledge (Q13), and 69.6% had used health-related apps, mini-programs or public accounts (Q15). The “Short video–social platforms + TV” class ranked second (1.99 ± 1.06), with corresponding proportions of 75.3%, 70.7%, and 53.0%, respectively.

By contrast, the “Offline village doctor/traditional channels (low-digital)” class showed the lowest level of digital health information engagement, with a mean engagement index of 0.47 ± 0.87 and proportions of 18.1%, 19.8%, and 9.3% for the three engagement behaviors, respectively. The “Mobile–WeChat/Douyin light-use” and “Village doctor/relatives + short video social” classes fell between these two extremes in terms of overall engagement level.

Statistical test results further indicated that differences across the five classes were significant for both the engagement index and the three individual engagement behaviors (Table 1). The Kruskal–Wallis χ^2^ for the engagement index was 261.48 (df = 4, *p* < 0.001), and the Pearson χ^2^ statistics for Q12, Q13, and Q15 were 209.74, 158.24, and 173.96, respectively (all *p* < 0.001). Based on these overall differences, pairwise Wilcoxon rank-sum tests with Holm adjustment showed that engagement levels in the “Omnichannel high-engagement” and “Short video–social platforms + TV” classes were significantly higher than those in the “Offline village doctor/traditional channels (low-digital)” and “Village doctor/relatives + short video social” classes, while the “Mobile–WeChat/Douyin light-use” class occupied an intermediate position in terms of the overall engagement index (Supplementary Table S2). Taken together, these results indicate that media-use classes centered on mobile internet, short-video platforms and social media had higher levels of digital health information engagement than lower-digital classes dominated mainly by offline and traditional channels.

#### 3.2.2 Soft-class robustness analysis using posterior-probability weighting

Given that LCA class assignment involves uncertainty in posterior probabilities, the hard-assignment approach based on modal class membership may underestimate within-class heterogeneity. We therefore conducted a robustness check using a soft-class approach. Specifically, the five-class posterior probabilities (post5_c1–post5_c5) were used as weights to calculate, for each class, the weighted mean of the engagement index and the three binary engagement behaviors, and class differences were compared using 95% bootstrap confidence intervals and pairwise differences derived from 2,000 bootstrap resamples (Supplementary Table S5).

The soft-class results showed that the ordering of the five classes on the engagement index was consistent with the hard-assignment analysis. The “Omnichannel high-engagement” class had the highest weighted mean (mean ≈ 2.24), followed by the “Short video–social platforms + TV” class (mean ≈ 1.90), the “Mobile–WeChat/Douyin light-use” class (mean ≈ 1.22), and the “Village doctor/relatives + short video social” class (mean ≈ 1.00), while the “Offline village doctor/traditional channels (low-digital)” class had the lowest weighted mean (mean ≈ 0.46; Supplementary Table S5A).

Pairwise comparisons further indicated that, for the engagement index, the weighted mean differences between most class pairs were significantly different from zero, with directions consistent with the hard-assignment analysis. For example, compared with the “Offline village doctor/traditional channels (low-digital)” class, the weighted differences in the engagement index for the “Mobile–WeChat/Douyin light-use,” “Omnichannel high-engagement,” “Short video–social platforms + TV,” and “Village doctor/relatives + short video social” classes were approximately 0.76, 1.78, 1.44, and 0.54, respectively, with bootstrap confidence intervals not crossing zero (*p*_boot < 0.001; Supplementary Table S5B). Overall, the consistency between modal hard-class assignment and posterior-probability weighting suggests that the observed class-related differences in digital health information engagement were not driven by the specific class-assignment method.

### 3.3 Regression-based associations with digital health information engagement

Table 5 presents the regression-based models examining cross-sectional associations among digital access and skills, perceived ease of understanding digital health content, lower operational difficulty, and digital health information engagement. All models adjusted for sex, age, education, monthly income, and media-use class.

**Table 5:** Regression-based models of cross-sectional associations with digital health information engagement.

Predictor	Model 1: engagement_index	Model 2a: Q16_rev	Model 2b: Q17_rev	Model 3: engagement_index
AccessSkills	0.327^***^ (0.046)	0.384^***^ (0.032)	0.465^***^ (0.035)	0.103^*^ (0.049)
Q16_rev	—	—	—	0.331^***^ (0.040)
Q17_rev	—	—	—	0.209^***^ (0.040)
Covariates	Yes	Yes	Yes	Yes
Media-use class	Yes	Yes	Yes	Yes

Values are unstandardized coefficients with HC3 robust standard errors in parentheses. Model 1 regresses engagement_index on AccessSkills. Models 2a and 2b regress Q16_rev and Q17_rev on AccessSkills, respectively. Model 3 regresses engagement_index on AccessSkills, Q16_rev, and Q17_rev. All models adjust for sex, age, education, monthly income, and media-use class. Q16_rev indicates perceived ease of understanding digital health content; Q17_rev indicates lower operational difficulty.

^*^p < 0.05, ^***^p < 0.001.

In Model 1, digital access and skills were positively associated with digital health information engagement (b = 0.327, robust SE = 0.046, *p* < 0.001). In Models 2a and 2b, digital access and skills were also positively associated with perceived ease of understanding digital health content (Q16_rev; b = 0.384, robust SE = 0.032, *p* < 0.001) and lower operational difficulty when using digital health information (Q17_rev; b = 0.465, robust SE = 0.035, *p* < 0.001).

When Q16_rev and Q17_rev were added to the engagement model in Model 3, both indicators remained positively associated with digital health information engagement. The coefficient was 0.331 for Q16_rev (robust SE = 0.040, *p* < 0.001) and 0.209 for Q17_rev (robust SE = 0.040, *p* < 0.001). The coefficient for digital access and skills was reduced from 0.327 in Model 1 to 0.103 in Model 3, while remaining statistically significant (robust SE = 0.049, *p* = 0.036). This corresponds to an attenuation of approximately 68.6% after accounting for perceived ease of understanding and lower operational difficulty.

These results suggest that digital access and skills were associated with higher digital health information engagement, and that this association was substantially attenuated after accounting for whether respondents found digital health content easier to understand and less difficult to operate. In substantive terms, the findings point to comprehension and operational ease as important correlates of digital health information engagement in this rural sample, rather than device or network access alone.

### 3.4 Regression-based associations with self-reported preventive behavior

Table 6 presents the regression-based models examining cross-sectional associations among digital health information engagement, attitudes toward health education, willingness to adopt new forms of health education, and the self-reported frequency of preventive behavior. All models adjusted for sex, age, education, monthly income, and media-use class.

**Table 6:** Regression-based models of cross-sectional associations with self-reported preventive behavior.

Predictor	Model 4: Q23_rev	Model 5a: Q26_rev	Model 5b: Q29_rev	Model 6: Q23_rev
Engagement_index	0.196^***^ (0.025)	0.156^***^ (0.023)	0.118^***^ (0.022)	0.138^***^ (0.026)
Q26_rev	—	—	—	0.132^**^ (0.046)
Q29_rev	—	—	—	0.318^***^ (0.050)
Covariates	Yes	Yes	Yes	Yes
Media-use class	Yes	Yes	Yes	Yes

Values are unstandardized coefficients with HC3 robust standard errors in parentheses. Model 4 regresses Q23_rev on engagement_index. Models 5a and 5b regress Q26_rev and Q29_rev on engagement_index, respectively. Model 6 regresses Q23_rev on engagement_index, Q26_rev, and Q29_rev. All models adjust for sex, age, education, monthly income, and media-use class. Q23_rev indicates the self-reported frequency of preventive behavior; Q26_rev indicates perceived helpfulness of health education; Q29_rev indicates willingness to adopt new forms of health education.

^**^p < 0.01, ^***^p < 0.001.

In Model 4, digital health information engagement was positively associated with self-reported preventive behavior (Q23_rev; b = 0.196, robust SE = 0.025, *p* < 0.001). In Models 5a and 5b, engagement was also positively associated with attitudes toward health education (Q26_rev; b = 0.156, robust SE = 0.023, *p* < 0.001) and willingness to adopt new forms of health education (Q29_rev; b = 0.118, robust SE = 0.022, *p* < 0.001).

When Q26_rev and Q29_rev were added to the model predicting Q23_rev in Model 6, both variables were positively associated with self-reported preventive behavior. The coefficient was 0.132 for Q26_rev (robust SE = 0.046, *p* = 0.004) and 0.318 for Q29_rev (robust SE = 0.050, *p* < 0.001). The coefficient for digital health information engagement was reduced from 0.196 in Model 4 to 0.138 in Model 6, while remaining statistically significant (robust SE = 0.026, *p* < 0.001). This corresponds to an attenuation of approximately 29.6% after accounting for attitudes toward health education and willingness to adopt new forms of health education.

These results indicate that respondents with broader engagement in digital health information also reported more frequent preventive behavior. This association was partly attenuated after accounting for attitudes and willingness, but it remained statistically significant. In substantive terms, the findings suggest that digital health information engagement, positive attitudes toward health education, and willingness to try new forms of health education were each associated with higher levels of self-reported preventive behavior in this rural sample.

### 3.5 Robustness analyses and exploratory interaction checks

Several robustness analyses and exploratory interaction checks were conducted to assess whether the main association patterns were sensitive to ordered-outcome specification, construction of the digital access and skills index, uncertainty in media-use class assignment, and selected sociodemographic grouping variables.

First, ordered-outcome sensitivity models showed the same direction of associations as the primary linear regression models. Digital access and skills remained positively associated with digital health information engagement, and the associations involving perceived ease of understanding, lower operational difficulty, engagement, attitudes/willingness and self-reported preventive behavior were substantively consistent with the primary models.

Second, when the composite AccessSkills index was replaced by its component indicators, internet-use frequency showed the clearest independent association with digital health information engagement (b = 0.096, robust SE = 0.039, *p* = 0.014), whereas device familiarity and perceived network coverage did not show independent associations after adjustment. In the same model, perceived ease of understanding (b = 0.333, robust SE = 0.041, *p* < 0.001) and lower operational difficulty (b = 0.201, robust SE = 0.041, *p* < 0.001) remained positively associated with engagement.

Third, the LCA-related soft-class analysis using posterior-probability weighting showed a class ordering of digital health information engagement consistent with the modal hard-class results, as reported above. Overall, these robustness analyses supported the same broad association pattern as the primary regression-based analyses, while also indicating that the composite AccessSkills index should be interpreted as a pragmatic summary of basic digital access and use conditions rather than as a full digital literacy scale.

Finally, exploratory interaction checks by age, education, and monthly income were also conducted using regression models. For the association between AccessSkills and digital health information engagement, no interaction term with age group, education level, or monthly income was statistically significant. For the association between digital health information engagement and self-reported preventive behavior, the interaction with age group was statistically significant (b = 0.141, robust SE = 0.044, *p* = 0.001), indicating a stronger association among middle-aged/older adults (≥41 years; slope = 0.262, *p* < 0.001) than among younger adults (18–40 years; slope = 0.121, *p* < 0.001). Interactions with education and monthly income were not statistically significant. Overall, these exploratory checks suggested limited heterogeneity across socioeconomic strata, with the main exception of age-related variation in the engagement–self-reported preventive behavior association.

## 4 Discussion

### 4.1 Overview of main findings

This study examined health-information media-use patterns, digital health information engagement, and self-reported preventive behavior among rural adults recruited from five selected counties/districts in Guizhou, China. The main contribution of the study is to show how differences in digital access and skills, comprehension and operational ease, engagement with digital health information, and attitudes/willingness toward health education are interrelated in a disadvantaged rural digital environment. Rather than treating digital health inequality as a matter of device or network access alone, the findings point to a broader pattern in which access, usability, understanding, engagement, and self-reported preventive behavior are closely linked.

First, the latent class analysis identified five media-use patterns that reflected different combinations of offline interpersonal/professional channels, traditional media, mobile/internet access, and social media platforms. The class-level results showed a clear gradient in digital health information engagement. Residents in the omnichannel high-engagement and short video–social platforms + TV classes reported the highest levels of engagement, whereas those in the offline village doctor/traditional channels (low-digital) class reported the lowest engagement. The soft-class analysis using posterior-probability weighting showed a similar ordering, suggesting that the observed class differences were not driven only by the specific hard-assignment method. These findings indicate that rural residents encounter health information within differentiated media environments, and that digitally connected and multi-platform environments are associated with broader engagement in digital health information.

Second, the regression-based analyses showed that digital access and skills were positively associated with digital health information engagement. Digital access and skills were also positively associated with perceived ease of understanding digital health content and lower operational difficulty. After these two variables were included in the engagement model, the coefficient for digital access and skills was substantially attenuated but remained statistically significant. This pattern suggests that digital access and skills are not simply linked to engagement in a direct access-based manner; rather, their association with engagement is closely related to whether residents can understand digital health content and use digital tools with fewer operational barriers. In rural digital health education, therefore, the practical value of digital access depends partly on whether access can be converted into usable and understandable health-information engagement.

Third, digital health information engagement was positively associated with the self-reported frequency of preventive behavior. Engagement was also positively associated with more positive attitudes toward health education and stronger willingness to adopt new forms of health education. After attitudes and willingness were included in the model, the association between engagement and self-reported preventive behavior was attenuated but remained statistically significant. This indicates that engagement with digital health information is linked not only to reported preventive behavior itself, but also to a broader attitudinal and motivational orientation toward health education. The results therefore support the relevance of engagement as a proximal correlate of self-reported preventive behavior, while also showing that attitudes and willingness account for part of this association.

Fourth, the robustness analyses generally supported the interpretation of the primary latent class and regression-based findings. Ordered-outcome sensitivity models, AccessSkills component analyses, and soft-class LCA analyses generally supported the same overall interpretation. Exploratory interaction checks further suggested limited heterogeneity across education and income groups, while the association between digital health information engagement and self-reported preventive behavior appeared stronger among middle-aged and older adults than among younger adults. Taken together, these findings should be interpreted as cross-sectional associations among field-feasible indicators rather than as evidence of causal mechanisms. They nevertheless provide a coherent empirical account of how media-use environments, digital access and skills, usability-related barriers, digital health information engagement, and self-reported preventive behavior are connected in this rural sample.

### 4.2 Theoretical and scholarly implications

The findings contribute to ongoing discussions on digital health equity, the digital divide, media-use ecology, and KAP-oriented health education research in three main respects.

First, the study extends digital health equity research by showing that rural digital health inequality should not be understood only through a simple access/no-access distinction. Existing digital inclusion and digital health equity research has emphasized that access to devices and networks, skills in using digital technologies, and the ability to benefit from digital applications constitute layered dimensions of digital inequality (8–14). The present findings are consistent with this layered view. In this rural sample, digital access and skills were associated with digital health information engagement, but this association was substantially attenuated after perceived ease of understanding and lower operational difficulty were included. This suggests that the practical value of digital access depends partly on whether residents can understand and use digital health information in a manageable way. This interpretation is also consistent with eHealth literacy research, which emphasizes the ability to search for, understand, evaluate, and use online health information (33–36), and with studies suggesting that differences in such abilities may translate into unequal health-related benefits (34, 35, 37–43).

Second, the study adds a media-use ecology perspective to rural digital health communication research. Rather than treating rural residents as a homogeneous audience or classifying them only by whether they use the internet, the latent class analysis identified differentiated media-use patterns combining village doctors, relatives and friends, television, mobile internet, WeChat, short-video platforms, and other channels. Prior research has emphasized that media exposure and media environments shape how individuals encounter, interpret, and act on health information (27–32). The present findings extend this perspective to a disadvantaged rural setting by showing that digital health communication is embedded in mixed information environments where offline trusted intermediaries, traditional media, and digital platforms coexist. The higher engagement observed among the omnichannel high-engagement and short video–social platforms + TV classes suggests that digital health engagement is shaped not only by individual skills, but also by the broader media environment through which residents encounter health information. This supports the need to move beyond single-platform measures and to examine how channel combinations structure opportunities for health-information engagement.

Third, the findings offer a cautious empirical extension of KAP-oriented thinking in a digital health education context. The KAP framework remains useful for organizing how information exposure, attitudes, and practice-related outcomes may be connected in health education research (20–26). In the present study, digital health information engagement was associated with both more positive attitudes/willingness toward health education and more frequent self-reported preventive behavior, and the engagement–behavior association was attenuated after attitudes and willingness were included. This pattern is compatible with the staged logic of KAP, but it should be interpreted as a cross-sectional association pattern rather than as confirmation of a causal knowledge–attitude–practice sequence. In this sense, the study uses the KAP framework as an organizing perspective for examining associations among engagement, attitudes/willingness, and self-reported preventive behavior, while recognizing the limits of cross-sectional data.

Taken together, these findings suggest that digital health inequality in disadvantaged rural settings is produced through the combined operation of media-use environments, digital access and skills, usability-related barriers, and engagement with health information. This is particularly relevant in rural Guizhou, where mountainous geography, uneven development, constrained grassroots health-service resources, and heterogeneous digital conditions may jointly shape how residents access and use health information (51–57). The study therefore contributes to digital public health research by shifting attention from infrastructure access alone to the conditions under which access becomes understandable, usable, and behaviorally relevant. It also highlights the value of combining media-use classification with regression-based association analyses to examine how rural residents are differently positioned within digital health communication environments.

### 4.3 Practical and policy implications

From a practical perspective, the findings suggest that efforts to promote digital health education and preventive health behavior in disadvantaged rural settings should move beyond infrastructure expansion alone. Expanding internet access and digital devices remains important, but the present findings indicate that access becomes practically meaningful only when residents can understand digital health content, operate digital tools with fewer difficulties, and engage with health information in ways that are connected with attitudes, willingness, and self-reported preventive behavior. This is consistent with broader evidence that digital health interventions in rural and under-resourced settings need to combine access improvement with usability, support, and context-sensitive implementation (5).

First, rural digital health education programs should strengthen basic digital access-and-use support, especially for residents who are less familiar with smartphones, online search, QR-code-based services, app-based health information, and short-video or public-account content. In practice, this may include hands-on demonstrations, repeated step-by-step assistance, and low-threshold training delivered through village clinics, township health centers, community activity spaces, or village committee settings. Such support should not be limited to one-time instruction; repeated practice and locally available help may be particularly important for middle-aged and older adults and residents with lower prior digital experience (73).

Second, digital health content and delivery formats should be designed to be easier to understand and easier to use. The association between digital access and skills and digital health information engagement was substantially attenuated after perceived ease of understanding and lower operational difficulty were included, suggesting that comprehension and usability are important practical barriers. Digital health education materials for rural residents should therefore use clearer language, shorter and more focused messages, visual or audio-visual explanation, local examples, and simplified navigation paths. For mini-programs, official accounts, and app-based tools, interface design should reduce unnecessary steps, avoid dense text, and provide clear prompts so that residents can move from information exposure to actual use with less operational burden (74, 75).

Third, interventions should not stop at information delivery alone. Digital health information engagement was associated with self-reported preventive behavior, and this association was attenuated after attitudes toward health education and willingness to adopt new forms of health education were included. This suggests that effective digital health education should combine information provision with follow-up explanation, reminders, practical encouragement, and opportunities for residents to ask questions or receive feedback. In rural settings, this may include linking online content with offline guidance from village doctors, township health workers, or community organizers, so that residents are not only exposed to health information but also supported in interpreting and applying it in daily preventive practices (74–76).

Fourth, preventive health communication in rural settings should be stratified according to residents' existing media-use patterns rather than delivered through a single uniform channel, consistent with context-sensitive and user-centered digital health implementation approaches (75–77). The five media-use classes identified in this study suggest different practical communication priorities.

For the “Offline village doctor/traditional channels (low-digital)” class, health communication should continue to rely on village doctors, township health centers, television, village-level announcements, printed materials and face-to-face explanation. For this group, digital intervention should begin with assisted use, such as helping residents scan QR codes, follow official accounts, watch short videos under guidance, or use simple mini-program functions in village clinics or community settings.

For the “Mobile–WeChat/Douyin light-use” class, communication can build on residents' existing use of mobile internet, WeChat and Douyin, but should avoid assuming deep digital engagement. Suitable strategies include short, clear WeChat messages, official-account articles with visual summaries, Douyin-style short videos, and simple reminders that direct users to trustworthy sources. Because this group has some digital access but relatively limited engagement breadth, interventions should focus on credibility, repetition, and low-effort participation rather than complex interactive functions.

For the “Short video–social platforms + TV” class, short-video platforms and television can be used as mutually reinforcing channels. Health education for this group may be delivered through short videos, myth-correction clips, village-level TV or screen broadcasts, and shareable platform content linked to official or local health-service sources. Because this class already shows relatively high engagement, communication strategies should emphasize content quality, platform credibility, and clear behavioral prompts, so that frequent media exposure can be connected with specific preventive actions.

For the “Omnichannel high-engagement” class, more interactive and participatory formats may be feasible. These residents already use multiple traditional, interpersonal and digital channels, and therefore may be suitable targets for online lectures, live Q&A sessions, community check-in activities, app/mini-program reminders, or peer-sharing tasks. This group could also serve as a bridge for community diffusion, for example by sharing credible health information within family networks, WeChat groups, or village-level communication spaces.

For the “Village doctor/relatives + short video social” class, interventions should combine offline trusted intermediaries with digital social and short-video content. Village doctors, family members and local community workers can help interpret and reinforce short-video or WeChat-based health messages, especially when residents encounter confusing or contradictory information online. For this group, the key is not to replace offline trust networks with digital platforms, but to connect them: digital content can provide repeated exposure, while village doctors and relatives can provide explanation, reassurance and practical guidance.

Finally, the exploratory interaction checks suggested that the association between digital health information engagement and self-reported preventive behavior may be stronger among middle-aged and older adults than among younger adults. This finding should be interpreted cautiously, but it implies that older rural residents should not be viewed only as digitally disadvantaged or passive recipients of health education. When provided with understandable content, usable tools, and appropriate support, digital health engagement among middle-aged and older adults may be especially relevant to reported preventive practices. Rural digital health programs should therefore avoid excluding older residents from digital interventions and should instead design age-sensitive, low-threshold forms of participation that combine digital channels with offline interpersonal support.

In practical terms, rural digital health interventions may be more useful when they combine five elements: basic digital skills support, comprehension-oriented content design, usability-oriented interface and delivery formats, follow-up guidance that connects information with everyday preventive action, and stratified communication strategies adapted to residents' existing media-use patterns. Such an approach is more likely to support digital health equity than strategies focused only on expanding devices, networks, or single-platform information dissemination.

### 4.4 Strengths and limitations

This study has several strengths. First, it draws on a relatively large adult sample recruited through fieldwork in five selected rural counties/districts in Guizhou, a setting where quantitative evidence on digital health education and digital health communication remains limited. Although the sample is not statistically representative, it provides a sizable field-based dataset for examining digital health information engagement among rural residents in a disadvantaged western Chinese context.

Second, the study combines latent class analysis with regression-based association models. The latent class analysis characterizes differentiated media-use patterns across offline interpersonal/professional channels, traditional media, mobile/internet access, and social media platforms, while the regression models examine how digital access and skills, comprehension and operational ease, engagement, attitudes/willingness, and self-reported preventive behavior are interrelated. This combined approach helps link residents' broader information environments with individual-level digital health information engagement and self-reported behavioral tendencies.

Third, the study used several robustness and sensitivity analyses to examine whether the main interpretation was sensitive to class assignment, model specification, and measurement choices. These included posterior-probability weighted soft-class analyses, ordered-outcome sensitivity models, component-level checks for the AccessSkills index, and exploratory interaction checks by age, education, and monthly income. These analyses do not remove the limitations of the cross-sectional design, but they provide additional information on the stability and boundaries of the main association patterns.

Several limitations should nevertheless be considered when interpreting the findings. First, the study is based on cross-sectional survey data. The analyses can identify associations among digital access and skills, comprehension and operational ease, engagement, attitudes/willingness, and self-reported preventive behavior, but they cannot establish temporal ordering or causal direction. For example, residents who report more frequent preventive behavior may already have stronger health motivation and therefore be more likely to engage with digital health information. Longitudinal, quasi-experimental, or intervention-based studies are needed to examine whether changes in digital access, usability support, or engagement lead to subsequent changes in preventive behavior.

Second, the study used a multistage non-probability sampling design involving purposive site selection, public-site recruitment, assisted questionnaire completion, local network circulation, and limited household outreach. To contextualize the obtained sample, the sample composition was compared with available 2020 census descriptors aggregated across the five sampled counties/districts (Supplementary Table S8). However, these comparisons are descriptive benchmarks only and do not establish population representativeness. Some groups may have been differentially represented; for example, residents who were more available in community settings or more willing to participate may have been overrepresented, whereas migrant workers temporarily away from the village, homebound residents, and residents with very low literacy or digital ability may have been underrepresented. The findings should therefore be interpreted as evidence from a non-probability adult sample recruited from selected rural sites, rather than as precise population estimates for rural Guizhou as a whole.

Third, several key constructs were measured using concise field-feasible indicators rather than full validated multi-item scales. The AccessSkills index provides a pragmatic summary of device familiarity, internet use, and perceived network coverage, but it does not capture the full complexity of digital literacy or eHealth literacy. The engagement index captures the breadth of digital health information use across three behaviors, but not frequency, duration, quality, credibility assessment, or depth of engagement. Similarly, perceived ease of understanding, operational difficulty, attitudes, willingness, and self-reported preventive behavior were measured using short proxy items. In particular, the preventive behavior outcome was measured using a single ordered item intended to capture broad self-reported preventive behavioral tendency/frequency, and it should not be interpreted as a comprehensive measure of specific preventive practices.

Fourth, all main variables were self-reported, which may introduce recall bias, social desirability bias, and common-method bias. Respondents may overreport desirable preventive behaviors or underreport difficulties in using digital tools, especially when questionnaires were completed with investigator assistance. Although assisted completion was necessary to reduce exclusion of older adults and residents with lower digital skills, it may also have influenced how some participants understood and responded to the questionnaire.

Fifth, the supplementary exploratory integrated observed-variable path model was used only as a descriptive check of the overall association pattern and is reported in Supplementary Tables S6, S7. Because its model-fit indices were mixed, it was not used to support substantive structural, pathway, mediation, mechanistic, temporal, or causal inference. The main interpretation rests on the latent class analysis and regression-based cross-sectional association models, together with the robustness and sensitivity analyses reported in the main text and Supplementary material.

Finally, although the models adjusted for sex, age, education, monthly income, and media-use class, residual confounding by unmeasured factors cannot be ruled out. Factors such as chronic disease status, family support, health-service quality, local health-program exposure, migration history, language or dialect barriers, and trust in village doctors or digital platforms may influence both digital health information engagement and preventive behavior. Future research would benefit from probability-based or more systematic sampling designs, richer validated measures of digital health literacy and preventive behavior, longitudinal or intervention designs, and more detailed analysis of how local media ecologies, health-service environments, and social support shape the uptake of digital health education in rural China.

### 4.5 Conclusions

This study examined media-use patterns, digital health information engagement, and self-reported preventive behavior among rural adults recruited from five selected counties/districts in Guizhou, China. The findings suggest that digital health inequality in disadvantaged rural settings is not limited to unequal access to devices, networks, or digital platforms. It is also reflected in whether residents can understand digital health content, use digital tools with fewer operational difficulties, and engage with health information in ways that are connected with attitudes, willingness, and self-reported preventive behavior.

The latent class analysis identified differentiated media-use patterns, showing that rural residents encounter health information through varying combinations of village doctors, relatives and friends, traditional media, mobile internet, WeChat, short-video platforms, and other channels. Residents in omnichannel and short-video/social-platform-centered classes reported higher levels of digital health information engagement, whereas those in the offline village doctor/traditional channels (low-digital) class reported the lowest engagement. These differences indicate that rural digital health communication should be adapted to residents' existing media-use environments rather than delivered through a single uniform channel.

The regression-based cross-sectional association models further showed that digital access and skills were positively associated with digital health information engagement, and that this association was substantially attenuated after accounting for perceived ease of understanding and lower operational difficulty. Digital health information engagement was also positively associated with self-reported preventive behavior, and this association was attenuated after accounting for attitudes toward health education and willingness to adopt new forms of health education. These findings should be interpreted as cross-sectional associations rather than evidence of causal mechanisms, but they highlight the importance of usability, comprehension, engagement, and attitudinal support in rural digital health education.

Overall, the study suggests that digital health education in underdeveloped rural settings should combine basic digital skills support, simplified and usable content design, trusted offline guidance, and differentiated communication strategies tailored to residents' media-use patterns. Improving rural digital health equity therefore requires not only expanding digital access, but also ensuring that digital health information is understandable, usable, trustworthy, and connected to residents' everyday preventive practices.

## Data Availability

The datasets generated and analyzed for this study are not publicly available in order to protect participant confidentiality in a rural small-community fieldwork setting. No names, national identification numbers, telephone numbers, biological samples, clinical records, or other directly identifying or medical information were collected at any stage. However, the individual-level survey records include contextual, sociodemographic, health-information-use, and self-reported behavioral variables from selected rural communities in Guizhou Province. Therefore, individual-level records are not publicly released. Aggregated data and analysis outputs underlying the main tables and figures can be provided by the corresponding author upon reasonable request and in line with local regulations and institutional requirements. Where applicable, any shared data would be further processed to exclude free-text responses and overly granular location information. The full questionnaire, including the Chinese original and English translation, is available in Supplementary Data Sheet 2. Requests to access the datasets should be directed to Yuxiao Lyu, lyuxiaobb@126.com.
