# Supplementary material for: Digital access, digital health information engagement, and self-reported preventive behavior among rural adults in Guizhou, China: media-use ecologies and cross-sectional associations
Source: Front Public Health. 2026 Jun 24;14:1794204. doi: 10.3389/fpubh.2026.1794204 (PMC13341684; doi:10.3389/fpubh.2026.1794204)
Supplement: Supplementary file 1 [file Data_Sheet_1.docx]

**Supplementary Material
Supplementary Data Sheet 1
Appendix Tables S1-S10**

**Supplementary Table S1. Overall descriptive statistics of key study variables (adult sample)**

N = 1,265 unless otherwise noted (missing values excluded per variable).

| **Variable** | **Scale** | **N** | **Mean ± SD** | **Median (IQR)** | **Min–Max** |
| --- | --- | --- | --- | --- | --- |
| Access & skills index (z) | z | 1265 | 0.000 ± 0.799 | -0.008 (-0.456–0.580) | -2.882–1.158 |
| Comprehension (Q16_rev) | 1-4 | 1265 | 2.847 ± 0.792 | 3 (2–3) | 1–4 |
| Less difficulty (Q17_rev) | 1-4 | 1265 | 2.965 ± 0.882 | 3 (3–4) | 1–4 |
| Engagement index (0-3) | 0-3 | 1265 | 1.353 ± 1.217 | 1 (0–3) | 0–3 |
| Helpfulness (Q26_rev) | 1-4 | 1265 | 3.308 ± 0.616 | 3 (3–4) | 1–4 |
| Willingness (Q29_rev) | 1-4 | 1265 | 3.289 ± 0.612 | 3 (3–4) | 1–4 |
| Self-reported preventive behavior (Q23_rev) | 1-4 | 1265 | 2.940 ± 0.884 | 3 (2–4) | 1–4 |
| Q12 used (binary) | 0/1 | 1265 | 0.532 ± 0.499 | 1 (0–1) | 0–1 |
| Q13 used (binary) | 0/1 | 1265 | 0.470 ± 0.499 | 0 (0–1) | 0–1 |
| Q15 used (binary) | 0/1 | 1265 | 0.351 ± 0.477 | 0 (0–1) | 0–1 |

Note. Q16_rev/Q17_rev/Q23_rev/Q26_rev/Q29_rev are reverse-coded so that higher scores indicate more favorable perceptions/behaviors. Engagement index is the sum of three binary items (Q12/Q13/Q15) and ranges 0-3.

**Supplementary Table S2. Descriptive statistics by media-use latent class (K = 5)**

**Panel A. Access, comprehension, operational ease and engagement by class**

| **Class (K = 5)** | **n** | **Access & skills (z)** | **Comprehension (Q16_rev)** | **Less difficulty (Q17_rev)** | **Engagement index** | **Median (IQR)** |
| --- | --- | --- | --- | --- | --- | --- |
| Village doctor/relatives + short video social | 158 | -0.140 ± 0.602 | 2.797 ± 0.582 | 2.829 ± 0.756 | 1.070 ± 1.097 | 1 (0–2) |
| Mobile–WeChat/Douyin light-use | 513 | 0.033 ± 0.677 | 2.827 ± 0.749 | 3.027 ± 0.762 | 1.177 ± 1.180 | 1 (0–2) |
| Offline village doctor/traditional channels (low-digital) | 182 | -0.910 ± 0.869 | 2.104 ± 0.868 | 1.995 ± 1.008 | 0.473 ± 0.869 | 0 (0–1) |
| Omnichannel high-engagement | 125 | 0.171 ± 0.671 | 3.136 ± 0.637 | 3.248 ± 0.700 | 2.248 ± 1.001 | 3 (2–3) |
| Short video–social platforms + TV | 287 | 0.521 ± 0.548 | 3.254 ± 0.609 | 3.422 ± 0.602 | 1.990 ± 1.054 | 2 (1–3) |

**Panel B. Attitudes, willingness, self-reported preventive behavior and engagement items by class**

| **Class (K = 5)** | **n** | **Helpfulness (Q26_rev)** | **Willingness (Q29_rev)** | **Self-reported preventive behavior (Q23_rev)** | **Q12 used** | **Q13 used** | **Q15 used** |
| --- | --- | --- | --- | --- | --- | --- | --- |
| Village doctor/relatives + short video social | 158 | 3.285 ± 0.564 | 3.209 ± 0.504 | 2.563 ± 0.917 | 43.0 | 38.6 | 25.3 |
| Mobile–WeChat/Douyin light-use | 513 | 3.255 ± 0.608 | 3.273 ± 0.562 | 2.914 ± 0.850 | 48.5 | 40.4 | 28.8 |
| Offline village doctor/traditional channels (low-digital) | 182 | 2.995 ± 0.683 | 2.896 ± 0.738 | 2.659 ± 1.008 | 18.1 | 19.8 | 9.3 |
| Omnichannel high-engagement | 125 | 3.440 ± 0.571 | 3.472 ± 0.530 | 3.048 ± 0.866 | 85.6 | 69.6 | 69.6 |
| Short video–social platforms + TV | 287 | 3.554 ± 0.511 | 3.530 ± 0.552 | 3.324 ± 0.665 | 75.3 | 70.7 | 53.0 |

Note. Values are within-class. Reverse-coded items are coded so that higher scores indicate more favorable conditions.

**Supplementary Table S3. Classification diagnostics for the five-class LCA solution**

| **Class label** | **Modal n** | **Modal %** | **Model-estimated class proportion** | **Mean maximum posterior probability** | **Mean classification uncertainty** |
| --- | --- | --- | --- | --- | --- |
| Mobile–WeChat/Douyin light-use | 513 | 40.60% | 37.40% | 0.803 | 0.197 |
| Offline village doctor/traditional channels (low-digital) | 182 | 14.40% | 14.30% | 0.921 | 0.079 |
| Omnichannel high-engagement | 125 | 9.90% | 10.50% | 0.956 | 0.044 |
| Short video–social platforms + TV | 287 | 22.70% | 24.10% | 0.842 | 0.158 |
| Village doctor/relatives + short video social | 158 | 12.50% | 13.70% | 0.779 | 0.221 |

Note. Modal n and modal % are based on maximum posterior-probability class assignment. Model-estimated class proportions are based on posterior membership probabilities from the selected five-class LCA model. Mean classification uncertainty was calculated as 1 minus the maximum posterior probability. Higher mean maximum posterior probability and lower classification uncertainty indicate clearer class assignment.

**Supplementary Table S4. Ordered-outcome sensitivity models for key regression-based associations**

| **Model** | **Outcome** | **Focal predictor** | **Estimate** | **SE** | **p** | **Comparison with primary model** |
| --- | --- | --- | --- | --- | --- | --- |
| M1 ordered | engagement_index | AccessSkills | 0.742 | 0.101 | <0.001 | Same direction |
| M3 ordered | engagement_index | AccessSkills | 0.236 | 0.112 | 0.034 | Same direction |
| M3 ordered | engagement_index | Q16_rev | 0.844 | 0.104 | <0.001 | Same direction |
| M3 ordered | engagement_index | Q17_rev | 0.606 | 0.101 | <0.001 | Same direction |
| M4 ordered | Q23_rev | engagement_index | 0.466 | 0.061 | <0.001 | Same direction |
| M6 ordered | Q23_rev | engagement_index | 0.333 | 0.063 | <0.001 | Same direction |
| M6 ordered | Q23_rev | Q26_rev | 0.367 | 0.104 | <0.001 | Same direction |
| M6 ordered | Q23_rev | Q29_rev | 0.840 | 0.107 | <0.001 | Same direction |

Note. Ordered-outcome sensitivity models were used because several outcomes were ordinal or index-based. The table reports only focal predictors corresponding to the primary regression-based association models; threshold parameters are omitted for readability. Results are interpreted by direction and statistical significance rather than direct coefficient comparability with linear models.

**Supplementary Table S5. Soft-class sensitivity analysis of digital health information engagement across media-use classes (posterior-probability weighted)**

This soft-class sensitivity analysis weights each respondent by posterior membership probabilities from the K = 5 latent class model. Pairwise differences are reported as the second class minus the first class in each comparison, with bootstrap 95% confidence intervals and bootstrap p-values.

**Panel A. Posterior-weighted class means**

| **Class** | **Class label** | **Effective n** | **Class proportion** | **Engagement index mean** | **Q12 used** | **Q13 used** | **Q15 used** |
| --- | --- | --- | --- | --- | --- | --- | --- |
| 1 | Mobile-WeChat/Douyin light-use | 472.9 | 37.4% | 1.224 | 50.1% | 42.3% | 29.9% |
| 2 | Offline village doctor/traditional channels (low-digital) | 181.3 | 14.3% | 0.459 | 17.4% | 19.0% | 9.4% |
| 3 | Omnichannel high-engagement | 133.4 | 10.5% | 2.238 | 85.2% | 69.6% | 68.9% |
| 4 | Short video-social platforms + TV | 304.3 | 24.1% | 1.900 | 72.4% | 66.8% | 50.8% |
| 5 | Village doctor/relatives + short video social | 173.1 | 13.7% | 0.997 | 40.7% | 36.5% | 22.4% |

**Panel B1. Pairwise differences in engagement index (0-3)**

| **Comparison** | **Difference** | **95% CI** | **p (bootstrap)** |
| --- | --- | --- | --- |
| Offline low-digital - Mobile-WeChat/Douyin light-use | -0.765 | [-0.897, -0.626] | <0.001 |
| Omnichannel high-engagement - Mobile-WeChat/Douyin light-use | 1.015 | [0.831, 1.188] | <0.001 |
| Short video/social + TV - Mobile-WeChat/Douyin light-use | 0.676 | [0.548, 0.800] | <0.001 |
| Doctor/interpersonal + short-video - Mobile-WeChat/Douyin light-use | -0.227 | [-0.368, -0.096] | <0.001 |
| Omnichannel high-engagement - Offline low-digital | 1.779 | [1.590, 1.970] | <0.001 |
| Short video/social + TV - Offline low-digital | 1.441 | [1.284, 1.571] | <0.001 |
| Doctor/interpersonal + short-video - Offline low-digital | 0.538 | [0.366, 0.694] | <0.001 |
| Short video/social + TV - Omnichannel high-engagement | -0.338 | [-0.524, -0.155] | <0.001 |
| Doctor/interpersonal + short-video - Omnichannel high-engagement | -1.241 | [-1.428, -1.025] | <0.001 |
| Doctor/interpersonal + short-video - Short video/social + TV | -0.903 | [-1.061, -0.751] | <0.001 |

**Panel B2. Pairwise differences in Q12 used phone/computer to obtain health information**

| **Comparison** | **Difference** | **95% CI** | **p (bootstrap)** |
| --- | --- | --- | --- |
| Offline low-digital - Mobile-WeChat/Douyin light-use | -32.7 pp | [-39.0, -26.5] pp | <0.001 |
| Omnichannel high-engagement - Mobile-WeChat/Douyin light-use | 35.1 pp | [28.4, 42.0] pp | <0.001 |
| Short video/social + TV - Mobile-WeChat/Douyin light-use | 22.2 pp | [17.2, 27.3] pp | <0.001 |
| Doctor/interpersonal + short-video - Mobile-WeChat/Douyin light-use | -9.4 pp | [-15.5, -3.1] pp | 0.002 |
| Omnichannel high-engagement - Offline low-digital | 67.8 pp | [60.1, 75.1] pp | <0.001 |
| Short video/social + TV - Offline low-digital | 55.0 pp | [48.5, 61.0] pp | <0.001 |
| Doctor/interpersonal + short-video - Offline low-digital | 23.4 pp | [15.6, 30.7] pp | <0.001 |
| Short video/social + TV - Omnichannel high-engagement | -12.9 pp | [-19.6, -6.3] pp | <0.001 |
| Doctor/interpersonal + short-video - Omnichannel high-engagement | -44.5 pp | [-52.3, -36.2] pp | <0.001 |
| Doctor/interpersonal + short-video - Short video/social + TV | -31.6 pp | [-38.4, -24.8] pp | <0.001 |

**Panel B3. Pairwise differences in Q13 used mobile health applications**

| **Comparison** | **Difference** | **95% CI** | **p (bootstrap)** |
| --- | --- | --- | --- |
| Offline low-digital - Mobile-WeChat/Douyin light-use | -23.3 pp | [-29.3, -17.4] pp | <0.001 |
| Omnichannel high-engagement - Mobile-WeChat/Douyin light-use | 27.3 pp | [19.3, 35.2] pp | <0.001 |
| Short video/social + TV - Mobile-WeChat/Douyin light-use | 24.4 pp | [18.9, 29.8] pp | <0.001 |
| Doctor/interpersonal + short-video - Mobile-WeChat/Douyin light-use | -5.8 pp | [-12.2, 0.5] pp | 0.076 |
| Omnichannel high-engagement - Offline low-digital | 50.6 pp | [41.5, 59.2] pp | <0.001 |
| Short video/social + TV - Offline low-digital | 47.7 pp | [40.6, 54.1] pp | <0.001 |
| Doctor/interpersonal + short-video - Offline low-digital | 17.5 pp | [10.0, 24.6] pp | <0.001 |
| Short video/social + TV - Omnichannel high-engagement | -2.9 pp | [-11.4, 5.5] pp | 0.502 |
| Doctor/interpersonal + short-video - Omnichannel high-engagement | -33.1 pp | [-42.0, -23.9] pp | <0.001 |
| Doctor/interpersonal + short-video - Short video/social + TV | -30.2 pp | [-37.3, -23.4] pp | <0.001 |

**Panel B4. Pairwise differences in Q15 participated in online health education content**

| **Comparison** | **Difference** | **95% CI** | **p (bootstrap)** |
| --- | --- | --- | --- |
| Offline low-digital - Mobile-WeChat/Douyin light-use | -20.5 pp | [-25.2, -15.4] pp | <0.001 |
| Omnichannel high-engagement - Mobile-WeChat/Douyin light-use | 39.0 pp | [30.5, 46.7] pp | <0.001 |
| Short video/social + TV - Mobile-WeChat/Douyin light-use | 20.9 pp | [15.4, 26.3] pp | <0.001 |
| Doctor/interpersonal + short-video - Mobile-WeChat/Douyin light-use | -7.5 pp | [-13.0, -1.9] pp | 0.012 |
| Omnichannel high-engagement - Offline low-digital | 59.5 pp | [50.5, 67.9] pp | <0.001 |
| Short video/social + TV - Offline low-digital | 41.4 pp | [35.5, 47.0] pp | <0.001 |
| Doctor/interpersonal + short-video - Offline low-digital | 12.9 pp | [7.2, 18.9] pp | <0.001 |
| Short video/social + TV - Omnichannel high-engagement | -18.1 pp | [-26.6, -9.6] pp | <0.001 |
| Doctor/interpersonal + short-video - Omnichannel high-engagement | -46.5 pp | [-54.9, -37.1] pp | <0.001 |
| Doctor/interpersonal + short-video - Short video/social + TV | -28.4 pp | [-35.0, -22.3] pp | <0.001 |

Note. Effective n equals the sum of posterior class membership probabilities. For Q12/Q13/Q15, differences are expressed in percentage points (pp). Class labels in pairwise comparisons are abbreviated for readability: “Offline low-digital” refers to Offline village doctor/traditional channels (low-digital), and “Doctor/interpersonal + short-video” refers to Village doctor/relatives + short video social.

**Supplementary Table S6. Fit indices for the exploratory integrated observed-variable path model**

| **Estimator** | **N** | **χ²** | **df** | **CFI** | **TLI** | **RMSEA** | **RMSEA 90% CI** | **SRMR** | **Interpretation** |
| --- | --- | --- | --- | --- | --- | --- | --- | --- | --- |
| MLR | 1,265 | 137.534 | 9 | 0.966 | 0.578 | 0.106 | 0.091-0.122 | 0.018 | Mixed fit; acceptable CFI/SRMR but elevated RMSEA and low TLI |
| WLSMV | 1,265 | 97.787 | 9 | 0.936 | 0.893 | 0.088 | 0.073-0.105 | 0.048 | Mixed but improved ordered-outcome specification |

Note. The integrated observed-variable path model was estimated as an exploratory summary of the overall association pattern. MLR used robust maximum likelihood with FIML for missing data. WLSMV treated ordinal/index-based variables as ordered where appropriate. Fit indices are reported for transparency and were interpreted alongside the regression-based primary analyses. The model was used to summarize the overall pattern of associations and was not treated as the primary basis for inference; no substantive structural, pathway, mediation, mechanistic, temporal, or causal inference was drawn from this exploratory model.

**Supplementary Table S7. Standardized coefficients from the exploratory integrated observed-variable path model**

| **Association** | **Std. β, MLR** | **p, MLR** | **Std. β, WLSMV** | **p, WLSMV** |
| --- | --- | --- | --- | --- |
| AccessSkills -> Q16_rev | 0.388 | <0.001 | 0.415 | <0.001 |
| AccessSkills -> Q17_rev | 0.422 | <0.001 | 0.444 | <0.001 |
| AccessSkills -> engagement_index | 0.067 | 0.032 | 0.052 | 0.097 |
| Q16_rev -> engagement_index | 0.216 | <0.001 | 0.344 | <0.001 |
| Q17_rev -> engagement_index | 0.151 | <0.001 | 0.229 | <0.001 |
| engagement_index -> Q26_rev | 0.308 | <0.001 | 0.499 | <0.001 |
| engagement_index -> Q29_rev | 0.235 | <0.001 | 0.509 | <0.001 |
| engagement_index -> Q23_rev | 0.190 | <0.001 | 0.398 | <0.001 |
| Q26_rev -> Q23_rev | 0.092 | 0.003 | 0.051 | 0.169 |
| Q29_rev -> Q23_rev | 0.220 | <0.001 | 0.216 | <0.001 |

Note. Coefficients are standardized estimates from the exploratory integrated observed-variable path model. The model was used to summarize the overall pattern of associations and was not treated as the primary basis for inference; no substantive structural, pathway, mediation, mechanistic, temporal, or causal inference was drawn from this exploratory model.

**Supplementary Table S8. Descriptive comparison between the analytic sample and aggregated 2020 census descriptors across the five sampled counties/districts in Guizhou**

| **Characteristic** | **Category** | **Sample (%)** | **Five-site census benchmark (%)** | **Comparability** |
| --- | --- | --- | --- | --- |
| Sex | Male | 48.9 | 51.4 | Direct |
| Sex | Female | 51.1 | 48.6 | Direct |
| Age | >60 in sample vs 60+ in census | 15.0 | 14.2 | Contextual only |
| Education | Mean years of schooling (approx.) | 9.73 | 8.03 | Contextual only |

Note. Census values are presented as descriptive benchmarks only to contextualize the sample composition. Because the study used a multistage non-probability sampling design and did not apply probability-based weighting or post-stratification adjustment, these comparisons should not be interpreted as evidence of population representativeness. Age and education comparisons should be interpreted cautiously because the survey included adults only, whereas the available census descriptors used different denominator structures.

Sources: Available county/district-level 2020 seventh population census bulletins or census summary pages for Dafang, Jinsha, Weining, Xixiu, and Xishui. Dafang: https://gzdafang.gov.cn/zwgk/dfxzfxxgkml/tjsj/tjgb/202106/t20210623_68790728.html; https://gzdafang.gov.cn/zwgk/dfxzfxxgkml/tjsj/tjgb/202106/t20210623_68790752.html; https://gzdafang.gov.cn/zwgk/dfxzfxxgkml/tjsj/tjgb/202106/t20210623_68790763.html. Jinsha and Weining: https://www.bijie.gov.cn/bm/bjstjj/zwgk/tjsj/tjgb/202106/t20210602_74711209.html; https://www.bijie.gov.cn/zwgk/zfsj/tjxx/tjgb/202106/t20210602_74919069.html; https://www.bijie.gov.cn/zwgk/zfsj/tjxx/tjgb/202106/t20210602_74919012.html. Xixiu: https://www.anshun.gov.cn/ksh/tjgb/202310/t20231013_82746449.html; https://www.anshun.gov.cn/ksh/tjgb/202310/t20231013_82746450.html; https://www.anshun.gov.cn/ksh/tjgb/202310/t20231013_82746451.html. Xishui: https://tjgb.hongheiku.com/rkpcgb/26865.html

**Supplementary Table S9. Questionnaire item wording, coding direction, analytic labels, and role in the revised analyses**

**Panel A. Questionnaire item wording and coding rules**

| **Code** | **Manuscript label** | **Item wording (CN / EN)** | **Response options (CN / EN)** | **Coding / direction** |
| --- | --- | --- | --- | --- |
| Q3 | Sex | 您的性别：What is your gender? | 男 \| 女Male \| Female | Sociodemographic covariate; treat as categorical. |
| Q4 | Age | 您的年龄段：What is your age group? | 18岁以下 \| 18~25 \| 26~30 \| 31~40 \| 41~50 \| 51~60 \| 60以上Under 18 \| 18–25 \| 26–30 \| 31–40 \| 41–50 \| 51–60 \| Over 60 | Covariate; age group for exploratory interaction checks: 18-40 vs >=41 years. |
| Q5 | Education | 您受教育的程度是？What is your highest level of education? | 未上过学 \| 小学 \| 初中 \| 高中 \| 中专 \| 大专 \| 本科及以上No formal schooling \| Primary \| Junior high \| High school \| Secondary vocational \| College \| Bachelor’s or above | Covariate; education group for exploratory interaction checks: junior high or below vs senior high or above. |
| Q6 | Monthly income | 您的月收入大概是多少？What is your approximate monthly income? | 2000元以下 \| 2000-5000元 \| 5000-10000元 \| 10000元以上Below 2,000 RMB \| 2,000–5,000 \| 5,000–10,000 \| Above 10,000 | Covariate; ordered/categorical as appropriate; income group for exploratory interaction checks: <5,000 CNY vs >=5,000 CNY. |
| Q7 | Health-information channels for LCA | 您平时通过什么途径获取健康知识？（可多选）Which sources do you usually use to obtain health knowledge? (Multiple choice) | 电视 \| 广播 \| 手机/网络 \| 村里的医生 \| 亲戚朋友 \| 其他Television \| Radio \| Mobile/Internet \| Village doctor \| Relatives/friends \| Other | Create 5 binary indicators (1=selected, 0=not): TV/radio/mobile-internet/village doctor/relatives-friends. Exclude “Other” from the 9 main LCA indicators. |
| Q8 | Device familiarity | 您觉得自己对使用手机或电脑的熟悉程度如何？How familiar are you with using a mobile phone or computer? | 非常熟悉 \| 比较熟悉 \| 一般 \| 不太熟悉 \| 完全不熟悉Very familiar \| Quite familiar \| Average \| Not very familiar \| Not familiar at all | Reverse-code so higher=better access/skills; then z-standardize and average with Q9–Q10 to form AccessSkills. (rev = 6 − raw) |
| Q9 | Internet-use frequency | 您平时会使用手机或电脑上网吗？How often do you use a mobile phone or computer to access the Internet? | 经常使用 \| 偶尔使用 \| 很少使用 \| 从来不用Often \| Sometimes \| Rarely \| Never | Reverse-code so higher=better access/skills; z-standardize. (rev = 5 − raw) |
| Q10 | Village network coverage | 您村里的网络覆盖怎么样？How is the Internet coverage in your village? | 覆盖很好 \| 覆盖一般 \| 覆盖不好 \| 没有网络Very good \| Average \| Poor \| No Internet | Reverse-code so higher=better access/skills; z-standardize. (rev = 5 − raw) |
| Q12 | Used phone/computer to obtain health information | 您有没有使用过手机或电脑获取健康信息？Have you ever used a phone or computer to obtain health information? | 使用过 \| 没有使用过Yes \| No | Binary: Yes=1, No=0. |
| Q13 | Used mobile health applications | 您是否使用过手机健康应用程序？Have you ever used mobile health apps? | 使用过 \| 没有使用过 \| 听说过但没有使用Yes \| No \| Heard of it but never used | Binary: Yes=1; No=0; Heard-but-never-used=0. |
| Q14 | Social media platforms for LCA | 您平时使用哪些社交媒体…了解健康信息？（可多选）Which social media platforms do you usually use to learn about health information? (Multiple choice) | 微信 \| 抖音 \| 快手 \| 微博 \| 其他 \| 不使用WeChat \| Douyin \| Kuaishou \| Weibo \| Other \| Do not use | Create 4 binary indicators (1=selected, 0=not): WeChat/Douyin/Kuaishou/Weibo. Exclude “Other” and “Do not use” from the 9 main LCA indicators. |
| Q15 | Participated in online health education content | 您曾经参加过在线健康教育课程或看过相关视频吗？Have you ever participated in online health education courses or watched related videos? | 参加过 \| 没有参加过 \| 听说过但没有参加Yes \| No \| Heard of it but never participated | Binary: Yes=1; No=0; Heard-but-never-participated=0. Then compute engagement_index as the sum of Q12/Q13/Q15 (range 0–3). |
| Q16 | Ease of understanding digital health content | …健康知识容易理解吗？How easy is it to understand the health knowledge explained in apps/social media/online courses? | 非常容易理解 \| 比较容易理解 \| 不太容易理解 \| 很难理解Very easy \| Easy \| Hard \| Very hard | Ordinal (4 levels); reverse-code so higher=better comprehensibility. (rev = 5 − raw) |
| Q17 | Lower operational difficulty | …获取健康信息时是否遇到困难？Do you encounter difficulties when using a phone/computer to obtain health information? | 没有困难 \| 有一点困难 \| 很多困难 \| 完全不会使用No difficulty \| A little \| A lot \| Cannot use at all | Ordinal (4 levels); reverse-code so higher=fewer difficulties. (rev = 5 − raw) |
| Q23 | Self-reported preventive behavior | 您平时会主动预防疾病吗？Do you take proactive measures to prevent diseases in daily life? | 经常 \| 偶尔 \| 很少 \| 从不Often \| Sometimes \| Rarely \| Never | Ordinal (4 levels); reverse-code so higher=more self-reported preventive behavior. (rev = 5 − raw) |
| Q26 | Perceived helpfulness of health education | 健康教育对改善生活质量有多大帮助？How helpful is health education in improving your quality of life? | 很大帮助 \| 一定帮助 \| 帮助不大 \| 没有帮助Very helpful \| Helpful \| Not very helpful \| Not helpful at all | Ordinal (4 levels); reverse-code so higher=more positive attitude. (rev = 5 − raw) |
| Q29 | Willingness to adopt new forms of health education | 更方便方式是否愿意尝试？If there are more convenient ways to obtain health information, are you willing to try them? | 非常愿意 \| 比较愿意 \| 不太愿意 \| 完全不愿意Very willing \| Willing \| Not very willing \| Not willing at all | Ordinal (4 levels); reverse-code so higher=stronger intention. (rev = 5 − raw) |

**Panel B. Analytic variables and role in the revised analyses**

| **Item / analytic variable** | **Manuscript label** | **Coding / direction** | **Role in revised analysis** |
| --- | --- | --- | --- |
| Q3 | Sex | Sociodemographic covariate; treat as categorical. | Covariate in regression-based association models |
| Q4 | Age | Covariate; age group for exploratory interaction checks: 18-40 vs >=41 years. | Covariate in regression-based association models; age group used in exploratory interaction checks |
| Q5 | Education | Covariate; education group for exploratory interaction checks: junior high or below vs senior high or above. | Covariate in regression-based association models; education group used in exploratory interaction checks |
| Q6 | Monthly income | Covariate; ordered/categorical as appropriate; income group for exploratory interaction checks: <5,000 CNY vs >=5,000 CNY. | Covariate in regression-based association models; income group used in exploratory interaction checks |
| Q7 | Health-information channels for LCA | Create 5 binary indicators (1=selected, 0=not): TV/radio/mobile-internet/village doctor/relatives-friends. Exclude “Other” from the 9 main LCA indicators. | Indicators for latent class analysis of media-use patterns |
| Q8 | Device familiarity | Reverse-code so higher=better access/skills; then z-standardize and average with Q9–Q10 to form AccessSkills. (rev = 6 − raw) | Component of AccessSkills; component-level robustness check |
| Q9 | Internet-use frequency | Reverse-code so higher=better access/skills; z-standardize. (rev = 5 − raw) | Component of AccessSkills; component-level robustness check |
| Q10 | Village network coverage | Reverse-code so higher=better access/skills; z-standardize. (rev = 5 − raw) | Component of AccessSkills; component-level robustness check |
| Q12 | Used phone/computer to obtain health information | Binary: Yes=1, No=0. | Component of engagement_index |
| Q13 | Used mobile health applications | Binary: Yes=1; No=0; Heard-but-never-used=0. | Component of engagement_index |
| Q14 | Social media platforms for LCA | Create 4 binary indicators (1=selected, 0=not): WeChat/Douyin/Kuaishou/Weibo. Exclude “Other” and “Do not use” from the 9 main LCA indicators. | Indicators for latent class analysis of media-use patterns |
| Q15 | Participated in online health education content | Binary: Yes=1; No=0; Heard-but-never-participated=0. Then compute engagement_index as the sum of Q12/Q13/Q15 (range 0–3). | Component of engagement_index |
| Q16 | Ease of understanding digital health content | Ordinal (4 levels); reverse-code so higher=better comprehensibility. (rev = 5 − raw) | Explanatory correlate in engagement models |
| Q17 | Lower operational difficulty | Ordinal (4 levels); reverse-code so higher=fewer difficulties. (rev = 5 − raw) | Explanatory correlate in engagement models |
| Q23 | Self-reported preventive behavior | Ordinal (4 levels); reverse-code so higher=more self-reported preventive behavior. (rev = 5 − raw) | Self-reported preventive behavior outcome |
| Q26 | Perceived helpfulness of health education | Ordinal (4 levels); reverse-code so higher=more positive attitude. (rev = 5 − raw) | Explanatory correlate in self-reported preventive behavior models |
| Q29 | Willingness to adopt new forms of health education | Ordinal (4 levels); reverse-code so higher=stronger intention. (rev = 5 − raw) | Explanatory correlate in self-reported preventive behavior models |
| AccessSkills | Digital access and skills | Higher = better digital access and skills; mean of standardized reverse-coded Q8-Q10 | Main predictor in regression models for engagement |
| engagement_index | Digital health information engagement | Range 0-3; sum of Q12, Q13 and Q15 | Engagement outcome in M1/M3; predictor in self-reported preventive behavior models |
| class5_n | Media-use latent class | Five-class modal assignment | Contextual covariate in regression-based association models |

Note. Item wording and response options follow the questionnaire / original coding table used in the analysis. Role descriptions refer to the revised regression-based association analyses. Questionnaire codes are retained for transparency and reproducibility, whereas the main text preferentially uses construct-based labels for readability.

**Supplementary Table S10. Additional robustness and exploratory interaction checks**

**Panel A. Component-level robustness for the AccessSkills index**

| **Predictor** | **Outcome** | **Estimate** | **Robust SE** | **p** | **Interpretation** |
| --- | --- | --- | --- | --- | --- |
| Q8_z | engagement_index | 0.015 | 0.039 | 0.698 | Device familiarity component; not independently significant after adjustment |
| Q9_z | engagement_index | 0.096 | 0.039 | 0.014 | Internet-use frequency component; clearest independent component association |
| Q10_z | engagement_index | 0.006 | 0.026 | 0.814 | Network coverage component; not independently significant after adjustment |
| Q16_rev | engagement_index | 0.333 | 0.041 | <0.001 | Ease of understanding remained positively associated with engagement |
| Q17_rev | engagement_index | 0.201 | 0.041 | <0.001 | Lower operational difficulty remained positively associated with engagement |

**Panel B. Exploratory interaction checks by age, education, and monthly income**

| **Association tested** | **Grouping variable** | **Interaction b** | **Robust SE** | **p** | **Reference slope** | **Comparison slope** | **Interpretation** |
| --- | --- | --- | --- | --- | --- | --- | --- |
| AccessSkills x age group predicting engagement_index | Age group | 0.086 | 0.085 | 0.307 | 18-40: 0.279 | >=41: 0.366 | No significant interaction |
| AccessSkills x education group predicting engagement_index | Education level | 0.079 | 0.085 | 0.355 | Junior high or below: 0.319 | Senior high or above: 0.398 | No significant interaction |
| AccessSkills x income group predicting engagement_index | Monthly income | -0.108 | 0.115 | 0.348 | <5000 CNY: 0.357 | >=5000 CNY: 0.249 | No significant interaction |
| engagement_index x age group predicting Q23_rev | Age group | 0.141 | 0.044 | 0.001 | 18-40: 0.121 | >=41: 0.262 | Significant interaction; association stronger in comparison group |
| engagement_index x education group predicting Q23_rev | Education level | -0.003 | 0.046 | 0.948 | Junior high or below: 0.200 | Senior high or above: 0.197 | No significant interaction |
| engagement_index x income group predicting Q23_rev | Monthly income | -0.003 | 0.050 | 0.957 | <5000 CNY: 0.197 | >=5000 CNY: 0.194 | No significant interaction |

Note. Panel A replaces the composite AccessSkills index with standardized component indicators. Panel B reports exploratory interaction checks testing whether two selected regression-based associations varied by age group, education level, or monthly income. Models used HC3 robust standard errors and adjusted for sex, relevant sociodemographic covariates, and media-use class. These analyses were exploratory and were not used as the primary basis for inference.
