# Supplementary material for: Digital access, digital health information engagement, and self-reported preventive behavior among rural adults in Guizhou, China: media-use ecologies and cross-sectional associations
Source: Front Public Health. 2026 Jun 24;14:1794204. doi: 10.3389/fpubh.2026.1794204 (PMC13341684; doi:10.3389/fpubh.2026.1794204)
Supplement: Supplementary file 2 [file Data_Sheet_2.docx]

Supplementary Data Sheet 2

Survey questionnaire (Chinese original and English translation)

# English version

Guizhou Digital Health Education Survey Questionnaire

Hello! In order to better understand residents' needs for health education and help more people gain health knowledge, we have designed this questionnaire. Please answer according to your actual situation. Thank you for your support!

1. Please specify your city. [Open-ended]

2. Please specify your local area (e.g., Shatu Town). [Open-ended]

3. What is your gender? [Single choice]

○ Male

○ Female

4. Which age group are you in? [Single choice]

○ Under 18

○ 18–25

○ 26–30

○ 31–40

○ 41–50

○ 51–60

○ Above 60

5. What is your highest level of education? [Single choice]

○ No formal schooling

○ Primary school

○ Junior high school

○ Senior high school

○ Secondary technical school

○ Junior college

○ Bachelor’s degree or above

6. What is your approximate monthly income? [Single choice]

○ Below 2,000 RMB

○ 2,000–5,000 RMB

○ 5,000–10,000 RMB

○ Above 10,000 RMB

7. Through which channels do you usually obtain health knowledge? (You may choose more than one) [Multiple choice]

□ Television

□ Radio

□ Mobile phone / Internet

□ Village doctor

□ Relatives and friends

□ Other (please specify): ______________________

8. How familiar do you think you are with using a mobile phone or computer? [Single choice]

○ Very familiar

○ Quite familiar

○ Average

○ Not very familiar

○ Not familiar at all

9. Do you usually use a mobile phone or computer to access the Internet? [Single choice]

○ Often

○ Occasionally

○ Rarely

○ Never

10. How is the Internet coverage in your village? [Single choice]

○ Very good coverage; can access the Internet at any time

○ Average coverage; sometimes the network is poor

○ Poor coverage; it is difficult to get online

○ No Internet access

11. Do you know what “digital health education” is? [Single choice]

○ Yes

○ No

12. Have you ever used a mobile phone or computer to obtain health information? [Single choice]

○ Yes

○ No

13. Have you ever used mobile health applications (for example, health codes, WeChat health services, step counters, etc.)? [Single choice]

○ Yes

○ No

○ I have heard of them but never used them

14. Which social media platforms do you usually use to learn about health information (such as WeChat, Douyin, Kuaishou, etc.)? (You may choose more than one) [Multiple choice]

□ WeChat

□ Douyin

□ Kuaishou

□ Weibo

□ Other (please specify): ______________________

□ I do not use social media for health information

15. Have you ever participated in online health education courses or watched related videos? [Single choice]

○ Yes

○ No

○ I have heard of them but have not participated

16. Do you think the health knowledge presented in these mobile apps, social media, or online courses is easy to understand? [Single choice]

○ Very easy to understand

○ Quite easy to understand

○ Not very easy to understand

○ Very difficult to understand

17. Do you encounter difficulties when using a mobile phone or computer to obtain health information? [Single choice]

○ No difficulties

○ Some difficulties (e.g., not familiar with the operations)

○ Many difficulties (e.g., do not really know how to use it)

○ I cannot use them at all

18. If there were training courses, would you be willing to learn how to use a mobile phone or computer to obtain health information? [Single choice]

○ Very willing

○ Quite willing

○ Not very willing

○ Not willing at all

19. How would you describe your own health status? [Single choice]

○ Very healthy

○ Generally healthy with minor problems

○ Occasionally ill

○ Often ill

○ Have a chronic disease (such as hypertension, diabetes, etc.)

20. How would you describe the health status of your family members (for example, parents, spouse, or children)? [Single choice]

○ All are healthy

○ Some have minor illnesses

○ Some are often ill

○ Some have chronic diseases (such as hypertension, diabetes, etc.)

21. Is it convenient for you to seek medical care? (For example, whether there is a clinic or hospital in or near your village) [Single choice]

○ Very convenient

○ Quite convenient

○ Not very convenient

○ Very inconvenient

22. How do you usually obtain medical advice? (You may choose more than one) [Multiple choice]

□ Go directly to a hospital

□ Ask the village doctor

□ Consult relatives and friends

□ Search for information online by myself

□ Other (please specify): ______________________

23. In your daily life, do you take actions to prevent disease on your own initiative? [Single choice]

○ Often take preventive actions (such as regular check-ups, healthy diet)

○ Sometimes take preventive actions

○ Rarely take preventive actions

○ Never take preventive actions

24. When do you usually obtain health information? (You may choose more than one) [Multiple choice]

□ When I am sick

□ When I feel unwell

□ I learn some health information every day

□ I occasionally look at health information

□ Other (please specify): ______________________

25. What do you think is the most convenient way for you to obtain health knowledge? (You may choose more than one) [Multiple choice]

□ Watching videos on a mobile phone

□ Listening to programs on the radio

□ Explanations from the village doctor

□ Posters on the village bulletin boards

□ Using social media platforms such as Douyin, Weibo, Kuaishou, Xiaohongshu, etc.

□ Other (please specify): ______________________

26. To what extent do you think health education helps improve your quality of life? [Single choice]

○ Very helpful

○ Somewhat helpful

○ Not very helpful

○ Not helpful at all

27. In your opinion, how widely has digital health education been promoted in your village? [Single choice]

○ Very widely promoted

○ Generally promoted

○ Not very widely promoted

○ Not understood at all

28. What kinds of health knowledge are you most interested in learning about? (You may choose more than one) [Multiple choice]

□ Prevention and treatment of common diseases

□ Healthy diet and nutrition

□ Exercise and health

□ Chronic disease management (such as diabetes, hypertension, etc.)

□ Maternal and child health

□ Mental health

□ Other (please specify): ______________________

29. If there were more convenient ways to obtain health information, would you be willing to try them? [Single choice]

○ Very willing

○ Quite willing

○ Not very willing

○ Not willing at all

30. What types of health education would you like the village to provide in the future? (You may choose more than one) [Multiple choice]

□ More health talks

□ Sending health tips via mobile phone text messages

□ Organizing online health courses

□ Broadcasting health information

□ Other (please specify): ______________________

31. Do you have any suggestions for future digital health education in your village? (Optional) [Open-ended]

_________________________________

32. Which of the following best describes your current region? [Single choice]

○ Dafang County

○ Jinsha County

○ Weining County

○ Hezhang County

○ Xishui County

○ Rural areas of Xixiu District, Anshun City

○ Baling Town, Xingren City

○ Zhijin County

○ Yantang Village, Jindingshan Town

33. What is your occupation? [Single choice]

○ Agricultural worker (engaged in agriculture, forestry, animal husbandry, fishery, etc.)

○ Self-employed (shop owner, e-commerce operator, service industry, etc.)

○ Student (senior high school student, university student, etc.)

○ Unemployed / retired (homemaker, unemployed person, retired older adult, etc.)

○ Other occupation (such as village cadre, teacher, etc.; please specify): ______________________

34. What is your ethnic group (nationality)? [Single choice]

○ Han

○ Yi

○ Miao

○ Buyi

○ Other (please specify): ______________________

# Chinese original

贵州数字健康教育调查问卷

您好！为了更好地了解大家对健康教育的需求，帮助更多人获得健康知识，我们设计了这份问卷。请您根据实际情况填写，感谢您的支持！

1. 请选择城市: [填空题] *

_________________________________

2. 请填写您所在的地区（如沙土镇）: [填空题] *

_________________________________

3. 您的性别： [单选题] *

○男 ○女

4. 您的年龄段： [单选题] *

○18岁以下 ○18~25 ○26~30 ○31~40

○41~50 ○51~60 ○60以上

5. 您受教育的程度是？[单选题] *

○未上过学

○小学

○初中

○高中

○中专

○大专

○本科及以上

6. 您的月收入大概是多少？[单选题] *

○2000元以下

○2000-5000元

○5000-10000元

○10000元以上

7. 您平时通过什么途径获取健康知识？（可多选） [多选题] *

□电视

□广播

□手机/网络

□村里的医生

□亲戚朋友

□其他（请说明）__________ _________________

8.  您觉得自己对使用手机或电脑的熟悉程度如何？[单选题] *

○非常熟悉

○比较熟悉

○一般

○不太熟悉

○完全不熟悉

9. 您平时会使用手机或电脑上网吗？[单选题] *

○经常使用

○偶尔使用

○很少使用

○从来不用

10. 您村里的网络覆盖怎么样？[单选题] *

○覆盖很好，随时可以上网

○覆盖一般，有时候网络不好

○覆盖不好，很难上网

○没有网络

11. 您知道什么是“数字健康教育”吗？[单选题] *

○知道

○不知道

12. 您有没有使用过手机或电脑获取健康信息？[单选题] *

○使用过

○没有使用过

13. 您是否使用过移动健康应用程序（例如健康宝、微信健康、步数记录等）？[单选题] *

○使用过

○没有使用过

○听说过但没有使用

14. 您平时使用哪些社交媒体（如微信、抖音、快手等）来了解健康信息？（可多选） [多选题] *

□微信

□抖音

□快手

□微博

□其他（请说明）__________ _________________

□不使用

15. 您曾经参加过在线健康教育课程或看过相关视频吗？

[单选题] *

○参加过

○没有参加过

○听说过但没有参加

16. 您觉得这些移动应用、社交媒体或者在线课程中讲解的健康知识容易理解吗？[单选题] *

○非常容易理解

○比较容易理解

○不太容易理解

○很难理解

17. 您在使用手机或电脑获取健康信息时是否遇到困难？[单选题] *

○没有困难

○有一点困难（如操作不熟悉）

○很多困难（如不会使用）

○完全不会使用

18. 如果有培训课程，您愿意学习如何使用手机或电脑获取健康信息吗？[单选题] *

○非常愿意

○比较愿意

○不太愿意

○完全不愿意

19. 您的健康状况如何？[单选题] *

○非常健康

○健康有小问题

○偶尔生病

○经常生病

○患有慢性病（如高血压、糖尿病等）

20. 您的家人（例如父母、配偶或孩子）的健康状况如何？[单选题] *

○都很健康

○有人有小病

○有人经常生病

○有人患有慢性病（如高血压、糖尿病等）

21. 您平时看病方便吗？（例如村里或附近是否有卫生所、医院等）[单选题] *

○非常方便

○比较方便

○不太方便

○非常不方便

22. 您通常会通过什么方式获得医疗建议？

[多选题] *

□直接去医院

□问村里的医生

□咨询亲戚朋友

□自己在网上查找

□其他（请说明）__________ _________________

23. 您平时会主动预防疾病吗？[单选题] *

○经常主动预防（如定期体检、健康饮食）

○偶尔会预防

○很少预防

○从不预防

24. 您通常会在什么时候获取健康信息？（可多选）[多选题] *

□生病的时候

□觉得身体不舒服的时候

□每天都会了解一些

□偶尔会看

□其他（请说明）__________ _________________

25. 您觉得获取健康知识最方便的方式是什么？（可多选）[多选题] *

□通过手机看视频

□通过广播听节目

□村里医生讲解

□村里宣传栏张贴的海报

□通过抖音、微博、快手、小红书等社交媒体获取

□其他（请说明）__________ _________________

26. 您认为健康教育对改善您的生活质量有多大帮助？[单选题] *

○很大帮助

○一定帮助

○帮助不大

○没有帮助

27. 您认为数字健康教育在您村里普及程度如何？[单选题] *

○很普及

○一般普及

○不太普及

○完全不了解

28. 您觉得现在有哪些健康知识是您最想了解的？（可多选）[多选题] *

□常见疾病的预防和治疗

□健康饮食和营养搭配

□运动与健康

□慢性病管理（如糖尿病、高血压等）

□妇幼保健

□心理健康

□其他（请说明）__________ _________________

29. 如果有更方便的健康信息获取方式，您是否愿意尝试？[单选题] *

○非常愿意

○比较愿意

○不太愿意

○完全不愿意

30. 您希望村里未来提供哪些形式的健康教育？（可多选）[多选题] *

□更多健康讲座

□通过手机发送健康小知识短信

□组织在线健康课程

□健康信息广播

□其他（请说明）__________ _________________

31. 您对村里未来开展数字健康教育有何建议？（可不填）

[填空题]

_________________________________

32. 请选择您所在的地区 [单选题] *

○大方县

○金沙县

○威宁县

○赫章县

○习水县

○安顺西秀区乡村

○兴仁市巴铃镇

○织金县

○金顶山镇堰塘村

33. 您的职业是： [单选题] *

○务农人员（从事农业、林业、牧业、渔业等工作）

○个体经营者（商店老板、电商经营者、服务业等）

○学生（在校高中生、大学生等）

○无业/退休人员（家庭主妇、失业者、退休老人等）

○其他职业（如村干部、教师等，请注明）__________ _________________

34. 您的民族是？ [单选题] *

○汉族

○彝族

○苗族

○布依族

○其他（请注明） _________________
